# Nitrogenous Compound Utilization and Production of Volatile Organic Compounds among Commercial Wine Yeasts Highlight Strain-Specific Metabolic Diversity

**DOI:** 10.1128/spectrum.00485-21

**Published:** 2021-07-21

**Authors:** William T. Scott, Oscar van Mastrigt, David E. Block, Richard A. Notebaart, Eddy J. Smid

**Affiliations:** a Department of Chemical Engineering, University of California, Davis, California, USA; b Food Microbiology, Wageningen University & Research, Wageningen, The Netherlands; c Department of Viticulture and Enology, University of California, Davis, California, USA; Broad Institute

**Keywords:** volatile organic compounds, HS-SPME/GC-MS, *Saccharomyces cerevisiae*, fermentation, wine, metabolic modeling

## Abstract

Genetic background and environmental conditions affect the production of sensory impact compounds by Saccharomyces cerevisiae. The relative importance of the strain-specific metabolic capabilities for the production of volatile organic compounds (VOCs) remains unclear. We investigated which amino acids contribute to VOC production and whether amino acid-VOC relations are conserved among yeast strains. Amino acid consumption and production of VOCs during grape juice fermentation was investigated using four commercial wine yeast strains: Elixir, Opale, R2, and Uvaferm. Principal component analysis of the VOC data demonstrated that Uvaferm correlated with ethyl acetate and ethyl hexanoate production, R2 negatively correlated with the acetate esters, and Opale positively correlated with fusel alcohols. Biomass formation was similar for all strains, pointing to metabolic differences in the utilization of nutrients to form VOCs. Partial least-squares linear regression showed that total aroma production is a function of nitrogen utilization (*R*^2^ = 0.87). We found that glycine, tyrosine, leucine, and lysine utilization were positively correlated with fusel alcohols and acetate esters. Mechanistic modeling of the yeast metabolic network via parsimonious flux balance analysis and flux enrichment analysis revealed enzymes with crucial roles, such as transaminases and decarboxylases. Our work provides insights in VOC production in wine yeasts.

**IMPORTANCE** Saccharomyces cerevisiae is widely used in grape juice fermentation to produce wines. Along with the genetic background, the nitrogen in the environment in which S. cerevisiae grows impacts its regulation of metabolism. Also, commercial S. cerevisiae strains exhibit immense diversity in their formation of aromas, and a desirable aroma bouquet is an essential characteristic for wines. Since nitrogen affects aroma formation in wines, it is essential to know the extent of this connection and how it leads to strain-dependent aroma profiles in wines. We evaluated the differences in the production of key aroma compounds among four commercial wine strains. Moreover, we analyzed the role of nitrogen utilization on the formation of various aroma compounds. This work illustrates the unique aroma-producing differences among industrial yeast strains and suggests more intricate, nitrogen-associated routes influencing those aroma-producing differences.

## INTRODUCTION

It has been widely recognized that yeast cell growth and overall wine fermentation performance are regulated by initial nitrogen levels within the grape must. Consequently, nitrogen limitation can induce sluggish or stuck fermentations (1–4). Ammonium and amino acids are the primary nitrogen sources used by yeast for general biosynthetic purposes by transferring the amine functional group (5, 6). Not only are yeast cell growth and fermentation completion influenced by the quality and amount of ammonia and amino acids in the grape must, the production of many crucial volatile organic compounds (VOCs) that are associated with desirable wine bouquet are also impacted (7–11). More specifically, these desirable VOCs are higher alcohols and their associated esters and fatty acids. The higher alcohols are products of the Ehrlich pathway, which uses branched-chain and aromatic amino acids as the substrates (12).

It has been shown that an inverse correlation exists between initial nitrogen levels (excluding at low initial nitrogen levels) and fusel alcohol concentrations (8, 13–16). Furthermore, it has been demonstrated in wine fermentations using S. cerevisiae that amino acids are directly involved in the formation of higher alcohols, esters, and fatty acids, and that these volatile organic compounds subsequently influence aroma attributes of wines (12, 17). Nonvolatile compounds, including glycerol, malic acid, and succinic acid, have also been shown to fluctuate depending on nitrogen concentration and source (18–20). Although VOC precursors produced via the Ehrlich pathway have been confirmed, various other amino acids such as alanine, lysine, glycine, histidine, and glutamine could potentially act as precursors or regulators of numerous metabolic pathways linked to aroma compound production. Moreover, surprisingly little is known about the relationship between the dynamics and timing of amino acid utilization and VOC production throughout grape must fermentation. Modulating desirable VOC yield in production strains will allow for valuable process advances in improving wine aroma bouquet, as well as increased flexibility in the production of specific aroma compounds for targeted types of wines. In addition, broader insights into aroma development could potentially lead to the introduction of these qualities into production strains with other desirable characteristics. Acetate esters and medium-chain fatty acid (MCFA) esters exhibit a more intricate relationship with initial nitrogen levels because of their biosynthetic routes of production. However, previous work has shown ethyl acetate is positively correlated with medium nitrogen levels (7, 8, 15, 21, 22). A commonly used practice in winemaking is to add nitrogenous compounds to avoid problem fermentations empirically. Although this heuristic method is moderately successful, its benefits are inconsistent, since the sole addition of inorganic nitrogen as well as improper supplementation of nitrogen have been revealed to negatively impact fermentation performance and aroma compound formation (10, 23). More precisely, low yeast assimilable nitrogen concentrations (YAN) can cause stuck fermentations and lead to higher H_2_S levels, while high YAN concentrations may cause greater turbidity, stimulate microbial instability, and facilitate the formation of unpleasant aromas (24–26). Thus, it would be most advantageous to be able to predetermine nitrogen levels and the timing of additions to a wine medium to achieve proper aroma character and wine styles, though this is not yet possible with current knowledge of the system, especially across a range of commercial yeast strains.

The numerous S. cerevisiae strains that are selected for winemaking differ immensely in their aroma production profiles (27–29). For example, a study that used two strains demonstrated the strain with the higher nitrogen requirement formed the higher ester concentration during fermentation of a Chardonnay must (30). Strain-dependent VOC production profiles could be due to variations in how yeast cells metabolize and utilize nitrogenous compounds. Another study, in which three yeast strains were examined in chemically defined media with different nitrogen compositions, indicated measurable differences in volatile and nonvolatile compounds, especially for the total amount of esters (31). Moreover, Miller et al. (29) showed strain-specific differences in the production of volatile esters in Chardonnay grape juice with various initial nitrogen levels. Although these studies were groundbreaking in illustrating strain-dependent behavior for several yeast strains under various environmental conditions, they lacked insight regarding the roles specific nitrogenous compounds play in the formation of VOCs during fermentation, as well as the impact of the complex metabolic background of strains.

Previous studies (29, 31, 32) have examined the dynamics of the relationship between nitrogen utilization of commercial S. cerevisiae strains and the production of VOCs, e.g., fusel alcohols and acetate esters. However, earlier studies may be incomplete, as it has been suggested that other nitrogen sources or nitrogen-involved metabolic pathways play a role in VOC formation in alcoholic fermentations such as beer (32, 33). Here, four industrial S. cerevisiae strains, Elixir, Opale, R2, and Uvaferm, were chosen for study because they were reported to possess a range of fermentation and aroma-producing capabilities. We then measured strain-specific behavior correlated with the production of key wine-associated VOCs. In order to further understand the role of nitrogen utilization in the various aroma-producing attributes among these strains, we applied partial least squares (PLS) regression. Moreover, since mechanistic understanding of the relation between amino acid consumption and VOCs production is currently lacking (34), we applied genome-scale metabolic modeling techniques.

## RESULTS

### Fermentations.

**FIG 1:**
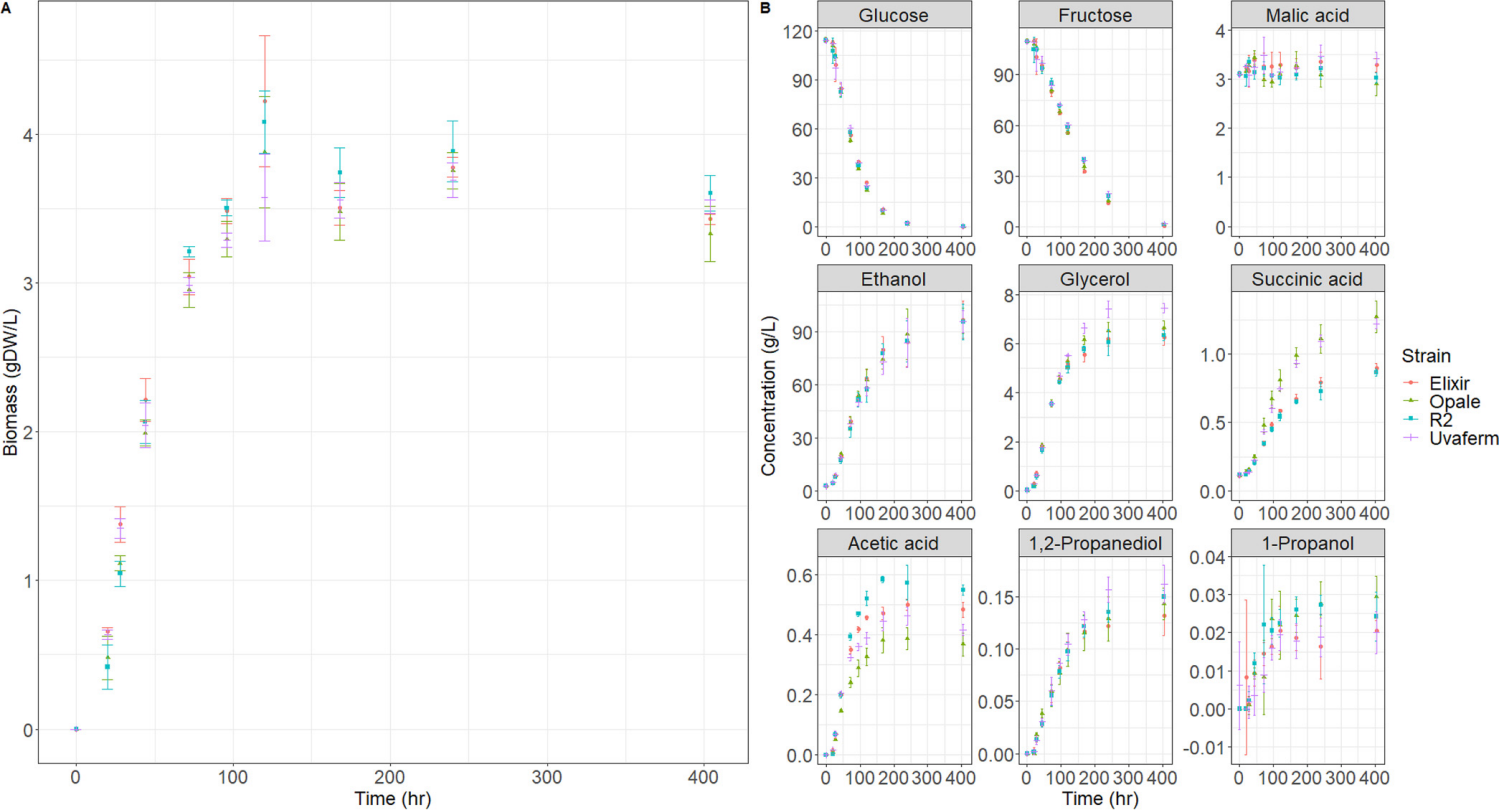
Growth, nutrient consumption, and metabolite production kinetics of the four yeast strains in MMM medium depicting dry cell weight (biomass) (A) and major metabolites (B) over the course of the fermentations. Error bars represent standard deviations (*n* = 3).

Fermentations were carried out for each of the S. cerevisiae strains in triplicate to evaluate nutrient utilization, i.e., ammonia, amino acids, and sugar consumption, as well as VOC (aroma) production capabilities across strains growing in the same enological medium. All fermentations were conducted under atmospheric conditions at a temperature of 20°C and were performed until completion (*t* = 404 h). Throughout the fermentation, cell biomass (estimated from optical density at 600 nm [OD_600_] measurements) and degrees Brix (°Bx) levels were monitored at 11 time points. Biomass growth, sugar utilization, and ethanol formation curves are shown (Fig. 1A).

All strains reached maximum biomass at approximately 120 h. Furthermore, all strains did not significantly differ in biomass concentrations (one-way ANOVA, *P* = 0.974). All cultures were fermented to completion (less than 4 g/liter residual sugar) (35). More specifically, fermentations performed with Uvaferm, R2, Opale, and Elixir strains resulted in total final sugar concentrations of 1.6 g/liter, 0.9 g/liter, 1.3 g/liter, and 0.3 g/liter, respectively. All the strains reached similar final ethanol concentrations ranging between 90 and 100 g/liter. The glycerol, malic acid, acetic acid, lactic acid, succinic acid, and 1,2 propanediol production were measured, as these compounds are vital to the sensory characteristics and stability of wines. Glycerol concentrations found in the cultures ranged from 6.3 g/liter for R2 to 7.5 g/liter for Uvaferm (Fig. 1B). These values are consistent with those found in wine (36). For all strains, malic acid levels were relatively constant throughout the fermentation at approximately 3 g/liter (Fig. 1B). Acetic acid production levels varied between the strains, with all of the final concentrations in the range of 350 mg/liter for Opale to 550 mg/liter for R2 (Fig. 1B). Succinic acid concentrations reached by the strains ranged from 0.8 g/liter for R2 to 1.3 g/liter for Opale (Fig. 1B). Final concentrations of 1,2 propanediol were relatively similar among the strains, ranging from 109 mg/liter for Elixir to 160 mg/liter for Uvaferm (Fig. 1B). One-way ANOVA revealed (95% confidence interval) that the production of acetic acid, malic acid, and succinic acid was significantly different among all of the strains (*P* < 0.05). In contrast, concentrations of the other six major metabolites (Fig. 1B) did not differ significantly among the strains (*P* > 0.05).

### Nitrogenous compound utilization and VOC production profiles during fermentation.

**FIG 2:**
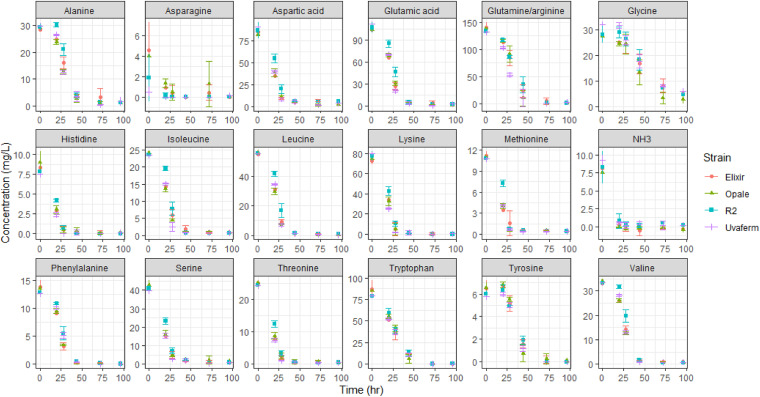
Amino acid and ammonia consumption of the four yeast strains in MMM medium showing the change in amino acid concentrations during fermentation. Error bars represent standard deviations (*n* = 3).

The concentrations of ammonia and amino acids were measured over the course of the fermentations (Fig. 2). Amino acid and ammonia consumption profiles deviated between all of the strains, illustrating strain-dependent behavior for some compounds such as alanine, glycine, asparagine, leucine, and phenylalanine. Moreover, concentrations of other amino acids such as serine, valine, lysine, methionine, and threonine, differed significantly across two or three strains. Overall, the level of consumption for 15 out of 20 amino acids studied differed significantly (ANOVA, *P* < 0.05). However, these and most variations in amino acid concentrations were observed at 20 and 28 h. As noticed (Fig. 2), it is evident that ammonia is consumed most rapidly, even before amino acids in the synthetic must. All of the amino acids were consumed during the fermentations except proline, which was not taken up and remained in the medium until the end of fermentation (data not shown).

Many amino acids were mainly consumed during the exponential cell growth stage of fermentation. Only some small amounts of Ala, Asn, Gly, and Met remained in the medium after 100 h, though all were utilized shortly after that. The consumption data reveal preferences of yeast for particular amino acids during utilization over the exponential growth phase. The consumption of the nitrogenous compounds can be separated into four groups based on the time at which 95% utilization occurs from the compound. Group I consists of the earliest-consumed compounds, where strain-dependent consumption of NH_3_, Asn, Lys, Met, Ile (Uvaferm only), and Thr (Uvaferm only) occurs, with 95% of their concentrations being drastically depleted by 28 h. Group II contains the subsequently preferred compounds, where 95% of the compound was consumed by 44 elapsed hours. These group II compounds are Arg, Gln, Ile, Leu, Phe, Ser, Thr, His, Asp, and Val. Group III consists of compounds such as Ala, Trp, and Tyr, which all show steady consumption until 95% of their concentrations were utilized by 72 h. A remaining group, group IV, was taken up after some initial delay (not consumed within the first 20 h) and did not have 95% utilization until after 96 h. This sole amino acid was Gly.

**FIG 3:**
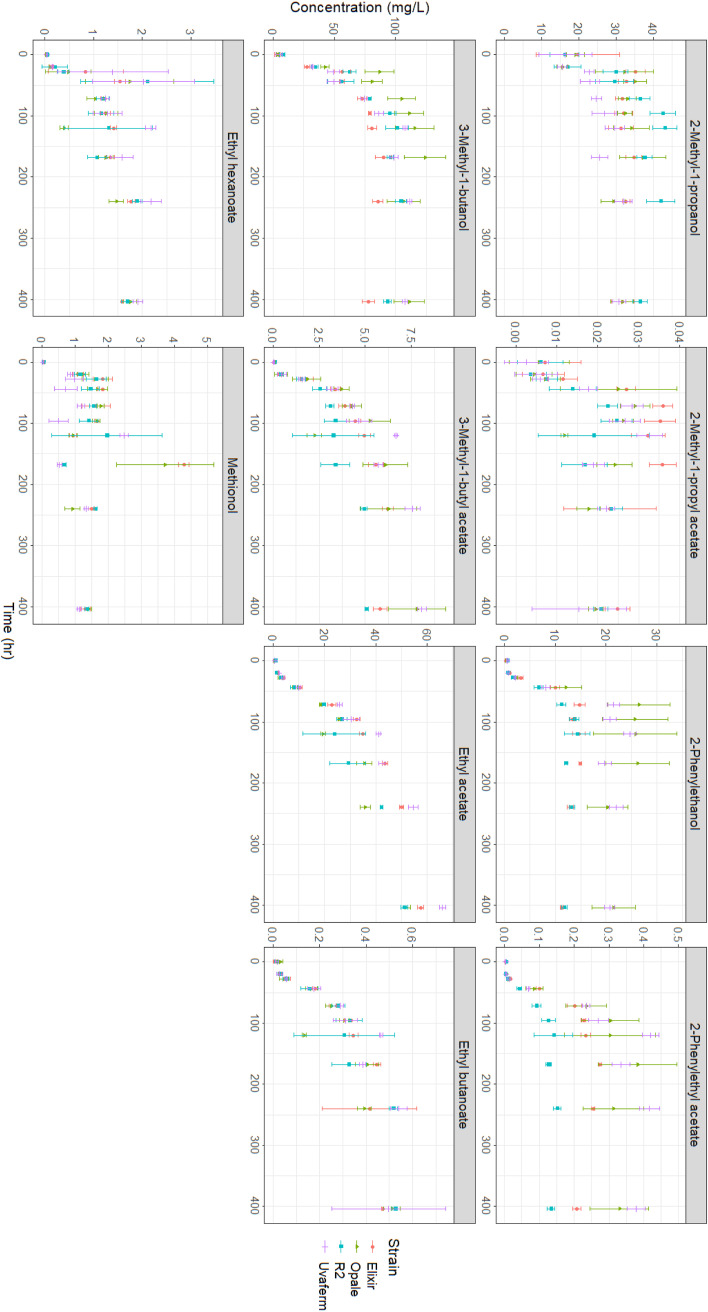
VOC (aroma) production of the four yeast strains in MMM medium showing the change in VOC concentrations over the course of the fermentations. Standard deviations are represented (*n* = 3).

To study the relationship between amino acid consumption and VOC formation during grape juice fermentation, 10 VOCs (aromas) were measured using headspace solid-phase microextraction gas chromatography mass spectrometry (HS SPME GC-MS) from various compound classes pertaining to different aroma properties throughout the fermentation process (Fig. 3). The compounds consisted of four fusel alcohols (isobutanol, isoamyl alcohol, 2-phenyl ethanol, and methionol), four acetate esters (ethyl acetate, isobutyl acetate, isoamyl acetate, and 2-phenyl ethyl acetate), and two ethyl esters (ethyl butanoate and ethyl hexanoate). The kinetics of the measured formation of VOCs were dependent on the yeast strain. In addition, the maximum and final concentration of VOCs varied significantly among the strains (ANOVA, *P* < 0.05). At 168 h of fermentation, the VOC concentrations among the strains were significantly different for eight VOCs, while two were the same. These compounds that did not differ significantly at the end of fermentation were 2-methyl-1-propanol and 2-methyl-1-propyl acetate.

All VOCs begin to form early in the fermentation process. Some compounds, such as 3-methyl 1-butanol and methionol, were even generated before 20 h, though nearly all VOC formation characteristically corresponds with yeast growth. Moreover, the maximum production rate of fusel alcohols and acetate esters occurred from 28 h to 96 h. Fusel alcohols were produced at the highest concentrations, with isoamyl alcohol, isobutanol, and 1-propanol showing the most rapid production earliest during fermentation. The acetate ester production rate began to decline for all of the strains after 96 h and became relatively stagnant after 168 h. Profiles for ethyl esters were similar to those observed for acetate esters. However, initial ethyl ester formation proceeded at a lower rate than acetate esters, and the maximum concentrations were much less than acetate esters. Overall, most VOCs showed stagnated production after 96 h, which indicates growth-dependent behavior. Some VOCs, in particular 2-methyl-1-propyl acetate, showed a decrease in concentration toward the end of fermentation, most likely due to volatilization. All final VOCs concentrations were found within the typical range found in wines (28).

**FIG 4:**
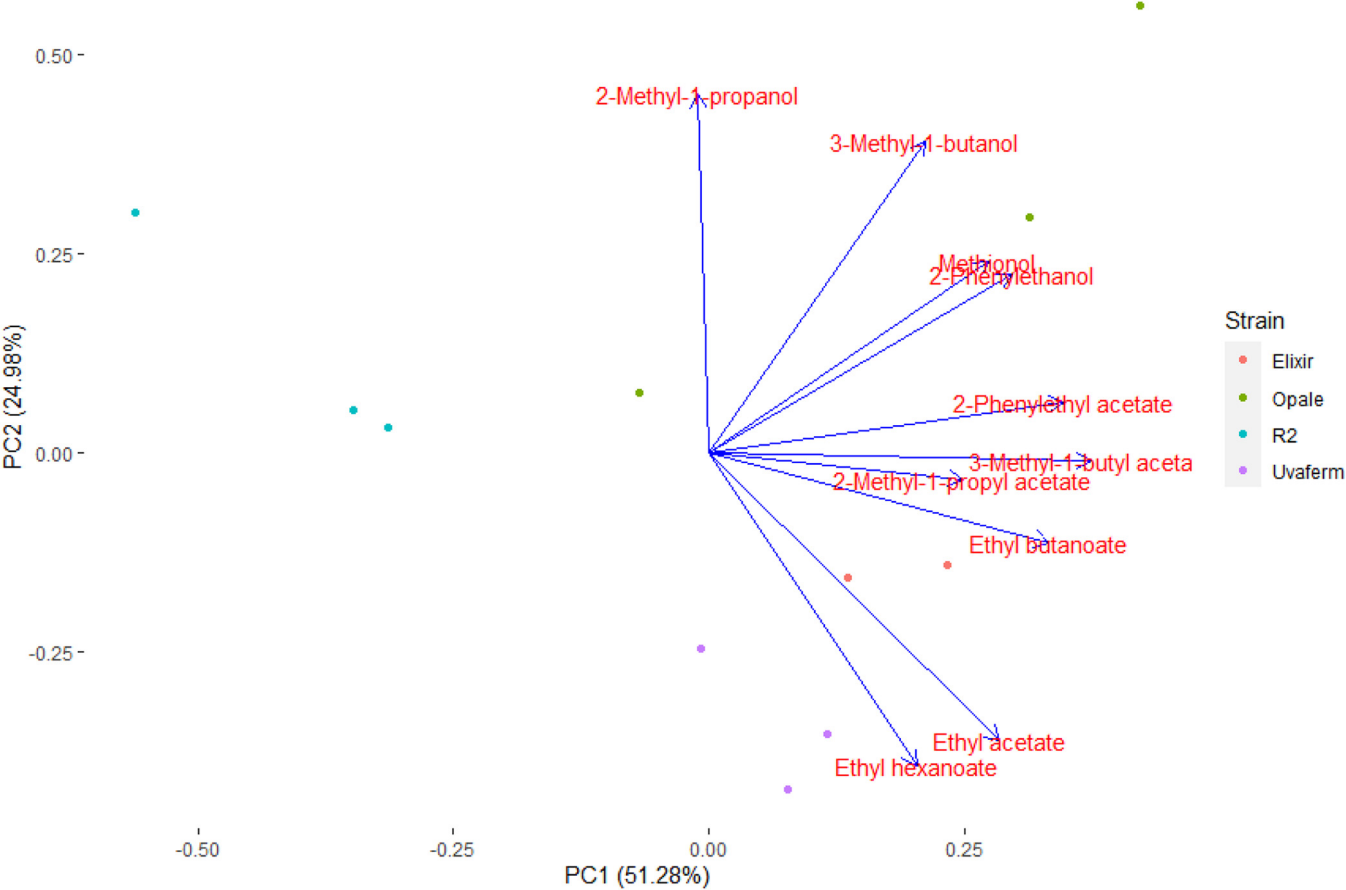
PCA scores and loadings plot of PC1 and PC2 derived from the volatile compounds produced by the yeast strains at *t* = 168 h.

In order to compare the results of volatile production by the strains, a principal-component analysis (PCA) of the VOCs was performed. From the PCA, 76.26% of the variance was explained by the first two principal components (PC) (PC1 = 51.25% and PC2 = 24.98%). As depicted, separation of the samples was achieved according to the yeast strains (Fig. 4). PC1 separated R2 from the other three strains, while PC2 separated Elixir, Opale, and Uvaferm strains. Although Lallemand (Lallemand, Montreal, Quebec) characterizes Uvaferm as a neutral aroma-imbuing strain, loadings for ethyl hexanoate and ethyl acetate were correlated with the Uvaferm strain, indicating relatively higher production for Uvaferm. Opale and R2 are described as conveying citrus, fruity, and floral aromas to wines from enhanced ester production and low H_2_S and SO_2_ formation. Nevertheless, higher loadings for fusel alcohols were correlated with the Opale strain along PC2, whereas loadings for the acetate esters were negatively correlated with the R2 strain, indicating relatively low acetate ester formation. The manufacturer claims that Elixir produces a wide array of beneficial fatty acid esters while limiting formation of acetate esters. As depicted, it appears Elixir has a correlation with ethyl butanoate and ethyl acetate (Fig. 4).

### Correlations of nitrogenous compound utilization with VOC formation.

One of the main hypotheses of this study asserted that utilization of ammonia and amino acids during fermentation contributed to the characteristic difference in the formation of VOCs among the yeast strains. First, ammonia and amino acids serve as an essential nitrogen source promoting good growth of the S. cerevisiae culture. Therefore, enhancing growth of yeast cultures leads to the overall increase of production of VOCs and VOC precursors. Second, it is known that specific amino acids are utilized and degraded which contribute to the synthesis, via the Ehrlich pathway, of fusel alcohols and subsequently to the production of acetate esters. However, other pathways related to lysine and glycine degradation, which have been shown to play a role in VOC formation in other types of fermentations, may be essential to enological fermentations as well. To evaluate this hypothesis and understand the causes of strain-dependent variation, the ammonia and amino acid utilization for all four yeast strains were measured and correlated with biomass formation and VOC production for 11 different aroma compounds during the exponential growth phase (Table 1).

**TABLE 1:** Summary of results from partial least-squares regression analysis

Response variable (predictor variable^*a*^)	No. of latent variables	RMSEC^*b*^	RMSECV^*b*^	*R* ^2^	*Q* ^2^	% Variance captured X-block	% Variance captured Y-block
Biomass concn. (nitrogen util.)	10	0.24	0.30	0.96	0.93	100.00	95.52
Fusel alcohol concn. (nitrogen util.)	5	47.25	53.11	0.87	0.83	100.00	87.02
1-Propanol concn. (nitrogen util.)	9	5.35	6.92	0.92	0.87	100.00	92.02
3 Methyl-1-butanol concn. (nitrogen util.)	7	35.56	40.57	0.82	0.77	100.00	82.32
2 Methyl-1-propanol concn. (nitrogen util.)	8	7.69	9.44	0.80	0.71	99.79	80.17
2 Phenylethanol concn. (nitrogen util.)	4	7.09	7.68	0.85	0.82	100.00	84.82
Methionol concn. (nitrogen util.)	8	0.64	0.73	0.61	0.50	100.00	61.01
Acetate ester concn. (nitrogen util.)	12	12.83	16.69	0.84	0.74	99.95	84.16
Ethyl acetate concn. (nitrogen util.)	11	11.10	14.85	0.85	0.73	99.97	84.48
3 Methyl-1-butyl acetate concn. (nitrogen util.)	12	1.51	2.06	0.85	0.74	99.91	85.43
2 Methyl-1-propyl acetate concn. (nitrogen util.)	14	0.01	0.01	0.85	0.74	100.00	85.42
2 Phenylethyl acetate concn. (nitrogen util.)	12	0.10	0.13	0.82	0.72	99.97	82.30
Fatty acid ethyl ester concn. (nitrogen util.)	4	0.73	0.81	0.77	0.72	100.00	77.19
Ethyl butanoate concn. (nitrogen util.)	11	0.12	0.16	0.86	0.75	99.98	86.09
Ethyl hexanoate concn. (nitrogen util.)	5	0.67	0.70	0.77	0.71	100.00	77.31
Total aroma concn. (nitrogen util.)	5	58.62	65.73	0.87	0.84	100.00	87.08

a “nitrogen util.” indicates ammonia and amino acid utilization.

b RMSEC, root mean squared error of correlation; RMSECV, root mean squared error in cross-validation.

In order to gain metabolic insight into how nitrogen utilization impacts characteristic VOC formation among the strains, the degree of the correlation between nitrogen consumption and VOC formation was evaluated using multivariate statistics. Since the data sets are high dimensional, partial least squared (PLS) linear regression was employed to correlate the contributions of ammonia and individual amino acid utilization to the production of biomass and VOCs by the yeast strains during fermentation. Statistical determination of the nitrogenous variables that contribute most to the variation in the fermentation kinetic data regarding VOCs was performed using interval-partial least squared (iPLS) regression analysis. iPLS was done using the ammonia and amino acid utilization of all four S. cerevisiae strains during the initial five time points covering the near complete uptake of the nitrogen during the first 96 h of fermentation. The biomass dry cell weight concentration, individual VOC concentration, each VOC class (i.e., fusel alcohol concentration, acetate ester concentration, and fatty acid ethyl ester concentration), and total VOC concentration were the response variables that comprised the Y-block in 16 different PLS regressions. The response variables contained concentration measurements corresponding to the same time points as the predictor variables. From the initial duration of the fermentation until 96 h, the ammonia and amino acid utilization data were input as the predictor variables used to develop the X-block in each of the 16 PLS regressions. Subsequently, the PLS regression analysis determined which variables in both the Y-block and the X-block contributed most to the variation in the data. Lastly, the PLS regression analysis indicated how the variables were correlated and then lumped the variables into a new latent variable (LV). Our summarized results show that nitrogen utilization is strongly correlated with biomass formation and production of each of the aroma compounds, except methionol, for which only moderate correlation was found (Table 1). The reason could be due to methionol production being controlled by sulfur uptake.

PLS regression was first conducted to determine how nitrogen utilization contributed to yeast growth (biomass formation) during the exponential growth phase in the fermentation. Table 1 summarizes the results from this analysis, where it lists that this model generated 10 LVs encompassing 100% of the variation in the nitrogen utilization data and 95.5 in the biomass concentration data. The PLS regression analysis yielded a correlation coefficient of *R*^2^ = 0.96, which signified a robust linear relationship between the measured biomass dry cell concentration versus the predicted biomass dry cell concentration according to the nutrient utilization of these yeast strains (Fig. S1 in the supplemental material). Cross-validation (CV) was performed in order to prevent the model from overfitting the data (i.e., model being applicable for the test observations and not being applicable for new observations) and to evaluate how the model would operate using a new set of data. CV correlation coefficients (*Q*^2^) were generated to illustrate the predictive strength of the model (37). The root mean squared error of correlation (RMSEC) for the biomass concentration as a function of nitrogen utilization was 0.24 g (dry weight) per liter of biomass, the root mean squared error in cross-validation (RMSECV) was 0.30 g (dry weight) per liter of biomass, and the *Q*^2^ value for this model was 0.93, which highlights that nitrogen utilization was an excellent predictor of the biomass concentration reached by these strains (Fig. S1).

A separate series of PLS regression analyses were performed subsequently for each of the VOCs, as well as classes of compounds, to investigate a correlation between the production of VOCs during the fermentations and nitrogen utilization of the yeast strains. Each of the models captured at least 99.5% of the variation in the nitrogen utilization data and at least 77% of the variation in the aroma concentration data were captured by each of the models except methionol (Table 1). In addition, the RMSEC and RMSECV for the individual aroma compounds and aroma compound classes as a function of ammonia and amino acid utilization are summarized in Table 1. The *R*^2^ value for each model for VOCs achieved an *R*^2^ value greater than 0.77, indicating nitrogen utilization was correlated with the production of each VOC. The only aroma compound that the PLS regression analysis showed to have a more modest correlation with nitrogen utilization was methionol. Partial least-squares regression modeling yielded eight LVs and generated a coefficient of determination (*R*^2^) of 0.61. The model cross-validation results indicate a *Q*^2^ of 0.5 and RMSEC and RMSECV values of 0.64 mg/liter and 0.73 mg/liter of methionol, respectively. Overall, these data point to a modest correlation between the methionol concentration and nitrogen utilization during fermentation. As a result, it is suggested that not only nitrogen utilization determines the production of methionol during fermentation and that there may be other processes that play a role in methionol production, such as sulfur utilization.

### Specific nitrogenous compounds associated with the formation of VOCs.

One of the core goals of this work was to improve understanding of the impacts of nitrogen utilization on VOC profile differences among four commercial wine yeast strains. By applying PLS to the data sets mentioned above, information about how specific nitrogen utilization variables correlate with each of the 16 response variable sets was obtained. This facilitated understanding of which nitrogenous compounds are responsible for aroma production and might offer clues about metabolic variations among the strains. The PLS models were able to explain the biomass formation and the production of each of the aroma compounds, excluding methionol, which was moderately predicted. Regression vector plots were created to assess the statistical weights of the original nitrogen predictor variables on the PLS models. In other words, regression vector plots were used to determine the degree to which the 18 original nitrogen variables were positively or negatively correlated with the response variable. These regression vector plots for PLS models predicting individual aroma compounds and classes of aroma compounds are provided in the supplemental material (Fig. S2), while a summary is provided in Table 2. In general, iPLS selection yielded more variables for ester models than for fusel alcohol models. All models shared many similar nitrogenous variables; however, most nitrogenous variable combinations were unique to each model for VOC predictions (Table 2).

**TABLE 2:** Summary of results from partial least-squares regression analysis vector plots of the predictor variables^*a*^

Model/variable	NH_3_	Ala	Arg/Gln	Asn	Asp	Glu	Gly	His	Ile	Leu	Lys	Met	Phe	Ser	Thr	Trp	Tyr	Val
Propan-1-ol	0	+	−	0	0	−	0	−	+	0	−	−	0	0	+	0	+	0
3 Methyl-1-butanol	0	0	−	0	−	−	+	0	0	+	+	0	0	0	0	0	+	0
2-Methylpropan-1-ol	−	−	0	0	0	−	+	−	0	0	+	−	+	+	−	−	0	0
2-Phenylethan-1-ol	0	0	−	0	−	0	+	0	0	0	0	0	0	0	0	0	+	0
Methionol	0	0	−	0	0	0	+	+	0	0	0	−	+	+	0	+	0	−
Ethyl acetate	0	0	−	+	+	−	+	−	+	−	+	−	−	0	0	0	+	−
3-Methylbutyl acetate	−	0	0	+	+	−	+	+	0	0	0	−	−	−	+	−	+	+
2-Methylpropyl acetate	0	0	+	+	+	−	+	−	0	−	+	0	−	+	−	+	+	+
2-Phenylethyl acetate	0	0	−	+	0	−	+	0	+	+	+	0	−	−	−	+	+	−
Ethyl butanoate	0	−	0	+	+	−	+	0	0	0	+	0	−	+	−	0	+	+
Ethyl hexanoate	0	0	0	0	0	0	0	0	+	−	−	−	0	0	0	0	+	0
Fusel alcohols	0	0	0	0	0	−	+	0	0	+	+	0	0	0	0	0	+	0
Acetate esters	0	+	−	+	0	+	+	0	+	0	+	0	−	+	−	+	+	−
Ethyl esters	0	0	0	0	0	+	0	0	+	0	−	0	0	0	0	0	+	0
Total aroma	0	0	0	0	0	−	+	0	0	+	+	0	0	0	0	0	+	0
Biomass	−	0	0	+	0	0	+	0	+	0	+	0	0	+	0	+	0	−

a 0, indicates variable not used; +, indicates positive correlation; −, indicates negative correlation. Note that the contribution magnitude of each nitrogen predictor variable to the respective PLS regression models is found in the supplemental material (Fig. S2) in the PLS vector plot.

The regression vector plots for all the yeast strains illustrate the degree to which the 18 original nitrogen predictor variables are correlated with the response variables (VOC and biomass concentrations) (Fig. S2). Furthermore, it is worth noting that the magnitude of the correlation coefficients for each nitrogen predictor variable was different across the PLS regression models (Fig. S2). The model for biomass growth indicated that asparagine, serine, glycine, lysine, isoleucine, and tryptophan were all positively correlated with biomass concentration, whereas, histidine, threonine, and valine are negatively correlated with biomass concentration (Table 2). Ammonia was shown to have a very slight negative correlation with biomass growth, with a correlation coefficient less than 0.1, but this contribution could be ascribed to measurement artifacts because more than one type of assay was needed to determine ammonia concentrations. The model for fusel alcohols indicated that Gly, Lys, Tyr, and Leu were positively correlated with fusel alcohol concentration, while Glu was negatively correlated with fusel alcohol concentration (Table 2). Ala, Asp, Ile, Ser, and Trp were also found to be positively correlated with acetate ester concentration, while Val, Arg, Gln, and Phe were negatively correlated with acetate ester concentration. Lastly, the ethyl esters model indicated that Glu, Ile, and Tyr were positively correlated with ethyl ester concentration, whereas Lys was negatively correlated with ethyl ester concentration.

A summary of the results from all the regression vector plots highlights correlative behavior of the nitrogen variables for predicting individual VOC production (Table 2). The regression vector plots revealed some patterned behavior regarding correlation found for particular nitrogenous compounds. Ammonia, methionine, and glutamic acid were only negatively correlated with the prediction of aroma compound production. Furthermore, arginine and glutamine negatively correlated with the prediction of most fusel alcohols and acetate esters, except 2-methyl propyl acetate, which was positively correlated. Conversely, some nitrogen variables were only positively correlated with aroma production, meaning that a higher consumption of these nitrogen compounds leads to higher aroma concentrations. These nitrogen variables were asparagine, isoleucine, and tyrosine. Glycine positively correlated with all aroma compounds except 1-propanol and ethyl hexanoate, and aspartic acid positively correlated with all acetate esters except 2-phenylethyl acetate. Nitrogen variables that were positively correlated with the total aroma model were glycine, leucine, lysine, and tyrosine. Lysine positively correlated with many fusel alcohols and acetate esters. The results for the remaining nitrogen variables were mixed concerning the prediction of aroma compound.

### Parsimonious flux balance analysis and flux enrichment analysis.

**FIG 5:**
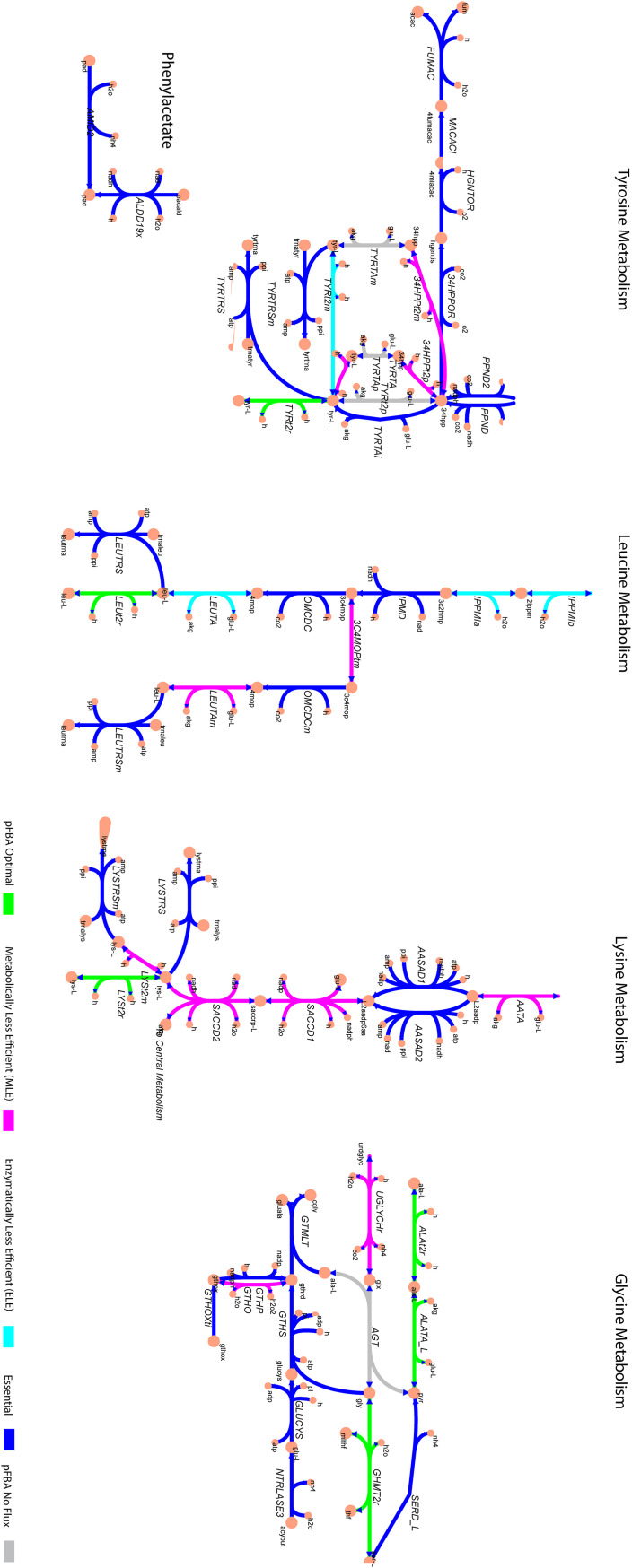
Key S. cerevisiae metabolic pathways illustrating reaction classes from applying parsimonious flux balance analysis. All reactions and metabolites shown in the figure contain Biochemical Genetic and Genomic (BiGG) database identifiers (http://bigg.ucsd.edu/).

To test which metabolic pathways are crucial in VOC production, we applied parsimonious flux balance analysis (pFBA) and flux enrichment analysis (FEA) with fermentation data used as constraints. The pFBA analysis revealed many essential genes/reactions for nutrient uptake and turnover, including amino acids, and VOC production (Table 3). After excluding transport reactions and nonenzyme-associated reactions, there are 1,047 essential reactions and 259 essential reactions are associated with amino acid degradation pathways, representing 24.7% of the essential reactions. Besides essential reactions, nonessential pFBA optimal (the most efficient routes to produce the observed metabolites even though others might be possible) and less efficient metabolic reactions (ELE and MLE reactions, i.e., ones that require more enzymatic steps than the minimum or lower growth rate than the maximum, respectively), are related to amino acid degradation and VOC-forming pathways (Fig. 5). The activity of less-efficient reactions may point to a trade-off between growth and VOC production, i.e., when VOCs are produced, less efficient reactions must also occur in context of growth.

**TABLE 3:** Summary of the essential reactions and the flux enrichment analysis results of yeast model subsystems

GSMM metabolism group^*a*^	No. of essential reactions	Enriched set size	*P* value
Sterol metabolism	36	49	4.80E−31
Tyrosine tryptophan and phenylalanine metabolism	32	44	1.23E−26
Complex alcohol metabolism	33	41	3.13E−24
Arginine and proline metabolism	23	33	1.79E−18
Methionine metabolism	16	20	1.40E−10
Glycine and serine metabolism	18	19	4.96E−10
Threonine and lysine metabolism	14	19	4.96E−10
Valine leucine and isoleucine metabolism	17	19	4.96E−10
Glycolysis and gluconeogenesis	19	22	1.06E−09
Pyruvate metabolism	17	20	1.40E−09
Glutamate metabolism	12	17	5.94E−09
Other amino acid metabolism	5	13	7.13E−07
Glycerolipid metabolism	9	12	2.28E−06
Cysteine metabolism	10	10	2.24E−05
Histidine metabolism	10	11	5.89E−05
Alanine and aspartate metabolism	8	9	6.88E−05
Phospholipid metabolism	7	8	0.00021
Fructose and mannose metabolism	5	6	0.00186
Citric acid cycle	10	10	0.00198
Glutamine metabolism	4	4	0.01578

a GSMM, genome-scale metabolic model.

In order to test whether the essential genes are part of particular metabolic pathways (or subsystems) by chance, we employed flux enrichment analysis (FEA) to identify statistically significantly enriched subsystems with essential reactions (38). FEA highlights tyrosine, glycine, lysine, and leucine metabolism and complex alcohol metabolism (fusel alcohols) as the most significant parts of metabolism, i.e., enriched with essential genes (Table 3). Thus, FEA supported the results from our PLS regression analysis. The above pFBA and FEA outcomes are based on the experimental data of strain Uvaferm and it should be noted that the outcomes are independent of the strain and thus illustrate a fundamental systems-level property of yeast metabolism (see Table S3 for an essential reaction list). We also found that sterol (lipid metabolism), arginine, and methionine (sulfur) metabolism play significant roles (*P* < 1 E−10). This result could indicate substantial influence coming from lipid metabolism and sulfur metabolism. Despite potential influence from other parts of metabolism on VOC formation, our statistical and metabolic modeling results firmly indicated that amino acid utilization routes such as leucine, glycine, lysine, and tyrosine induce VOC production (Fig. 5). We also observed within these amino acid utilization routes the essential reactions and less efficient routes (MLE and ELE), including an example VOC (2-phenylethyl acetate) (Fig. 5). Interestingly, it was observed that transaminase- (e.g., TYRTAi) and decarboxylase-associated reactions (e.g., OMCDC) were essential, indicating that Ehrlich or amino acid degradation pathways are involved in VOC formation. Nevertheless, other reactions were also found to be essential, such as reactions associated with acetate (e.g., FUMAC), suggesting to other routes influence VOC formation as well.

## DISCUSSION

Fermentations were performed with four commercial S. cerevisiae strains with diverse aroma bouquet-producing potential and growth characteristics under identical experimental conditions to analyze the nitrogenous compounds that correlated with yeast cell growth and aroma production. In agreement with previous studies (10, 29, 38, 39), these experimental results illustrate significant differences in volatile and nonvolatile production depending on the applied yeast strain. In most cases, the fermentations performed with the Opale strain had the highest maximum and final nonvolatile and volatile concentrations. In contrast, the fermentations conducted using the R2 strains had the lowest maximum and final nonvolatile and volatile concentrations. However, normalizing the nonvolatile and volatile concentrations to the biomass formed caused the differences among the strains to be less evident (data not shown). Furthermore, when examining the product specifications from Lallemand (Lallemand, Montreal, Quebec), it was presumed that the Uvaferm strain, which is a neutral aroma producer, would generate the least volatile compounds under the same medium conditions relative to the other strains. Our results suggested, as shown in previous studies, that R2, which, compared with Opale, produces less fusel alcohols and isoacids throughout the fermentation, might more effectively regulate carbon flux (less efficient usage of nitrogen) over time. Moreover, this aspect could be related to nitrogen causing less excretion of excess carbon from core carbon cellular pathways (39).

Contrary to VOC product formation, nutrient consumption behavior of nitrogen and sugar compounds was similar among the strains. It was observed that nitrogenous compound uptake began before the onset of growth in which ammonium was consumed first, similar to what was found in a previous study (40). Following ammonia consumption, there was a preference for specific amino acids (41). These preferred amino acids were Asp, His, Glu, Met, and Lys, all consumed within 44 h. These were also previously seen as the first-consumed amino acids in media with the same YAN conditions (42, 43). This preference in utilization of particular amino acids is thought to be linked to nitrogen catabolite repression (NCR) of amino acid transport permeases (24, 44). Many of the amino acids were consumed during the first 96 h of fermentation. However, some showed interesting kinetic behavior. Ala, Asn, and Glu showed a slight uptick in concentration during the stationary growth phase, which could be attributed to excretion before autolysis, as has been seen previously (45), though there are some differences regarding the type of amino acids.

The results from PLS linear regression modeling indicated that the production of VOCs is a function of nitrogen utilization. Previous studies have demonstrated that having adequate YAN levels at the beginning of fermentation is essential in determining yeast cell growth and producing desirable levels of VOCs (8, 27, 46). The production of VOCs such as fusel alcohols and their associated acetate esters stems from the catabolism of branched-chain and aromatic amino acids via the Ehrlich pathway (47, 48). Furthermore, the availability of specific nitrogenous compounds early on during fermentation alters the transcriptional regulation of genes involved with higher alcohol production and the formation of esters in yeast (49, 50). Naturally, upon reaching the stationary growth phase, the rate of production of VOCs by S. cerevisiae begins to decline (29). It is understood that esters play a role in the survival of yeast in the esterification of toxic MCFAs, thus facilitating their diffusion through the plasma membrane (51).

By comparing the correlations of concentrations of all other VOCs with nitrogen utilization (*R*^2^ ≥ 0.77) on the one hand, and methionol concentration with nitrogen utilization (*R*^2^ = 0.61) on the other hand, it appears likely that nitrogen utilization is not the only significant factor influencing methionol production. The relatively low correlation for methionol formation could be due to the absence of causal relationships between the metabolic pathways of methionol biosynthesis and nitrogen metabolism. Methionol biosynthesis is also intricately linked to the sulfate reduction pathway and, thus, the relationship of sulfur uptake during fermentation (52). Hence, this could explain the variation in PLS model predictions with other fusel alcohols. Previous works have confirmed that the role of central carbon metabolism in the synthesis of higher alcohols is significantly akin to the contribution of amino acids (53). However, it is assumed there is a balance between the amount of α-ketoacids supplied by central carbon metabolism and the amount of α-ketoacids converted into amino acids for protein biosynthesis. Thus, over the course of nitrogen utilization, the corresponding flux of α-ketoacids related to the nitrogen utilization over time would be negligible compared to the flux originating from the central carbon metabolism (54). The α-ketoacid pool available would then remain unchanged and not affect the production of fusel alcohols.

The regression vector plots from both PLS regression models indicated numerous nitrogenous compounds in common among the models and the degree to which the nitrogen sources had influenced the models. The PLS models for total aroma production and biomass concentration indicated shared some nitrogenous compounds that are positively correlated with the formation of biomass and VOCs. These amino acids were glycine and lysine. Furthermore, for total aroma it was determined that the most significant positively correlated amino acids were glycine, lysine, leucine, and tyrosine. These findings corroborate what has been shown in a previous study, albeit with beer yeasts (32). This correlation is likely caused by the direct connection to the formation of some of the most significant fusel alcohols (isobutanol, isoamylol, and propanol) and their subsequent acetate esters (isobutyl acetate, isoamyl acetate, and propyl acetate) (55, 56).

pFBA was performed to determine the essential and metabolically feasible distribution of fluxes throughout the yeast metabolic network. By employing pFBA and FEA, the significance among essential reactions was observed for nitrogenous utilization pathways, as well as sterol, pyruvate, and glycerolipid metabolism. A similar approach has been taken previously with Escherichia coli to understand network effects of iron metabolism in triggering oxidative stress in Caenorhabditis elegans (57). For instance, our analysis illustrates the link lysine plays in the formation of VOCs. In particular, this link is implied from the modeled essentiality among fluxes through amino acid permeases, mainly LYP1-regulated permeases which have been suggested to steer higher alcohol and acetate ester formation during beer fermentations (33). The pFBA revealed many essential reactions in leucine utilization pathways through the use of permeases, transaminases, and decarboxylases, alluding to the Ehrlich pathway to produce VOCs (48). This result suggests, as shown by Yoshimoto and coworkers (58), that overexpressing genes such LEU and BAT within leucine metabolism can increase isoamyl alcohol and isoamyl acetate secretion.

## MATERIALS AND METHODS

### Yeast strains.

All four yeast strains, Uvaferm 43 (Uvaferm), Lalvin R2 (R2), Lalvin ICV Opale (Opale), and Vitilevure Elixir Yseo (Elixir), used in this study are Lallemand (Lallemand, Montreal, Quebec) commercial yeast strains. Uvaferm and R2 are Saccharomyces bayanus, which is a hybrid of Saccharomyces cerevisiae, Saccharomyces eubayanus, and Saccharomyces uvarum, while Opale and Elixir are S. cerevisiae var. *cerevisiae*. All yeast strains were obtained from the UC Davis Enology Culture Collection. Additionally, yeast strains were chosen to have different fermentation and aroma-producing characteristics under the conditions selected. For instance, R2 and Elixir are reported to be wine yeast strains that produce relatively large amounts of esters. In contrast, Uvaferm is reported to be more of an aroma-neutral strain and Opale is reported to impart an enhanced, complex character to wines (Lallemand, Montreal, Quebec). The strains were stored at −80°C in a 25% (vol/vol) glycerol solution according to the method of Amberg et al. (59). The strains were streaked for single colony isolation on yeast extract peptone dextrose (YEPD) agar plates, incubated at 25°C for 24 to 48 h until sufficient colonies formed, and stored at 4°C for no longer than 30 days.

### Growth, fermentation media, and culture conditions.

MMM synthetic grape juice medium (220 g sugar/liter; 22.0 °Bx), with a 1:1 mixture of glucose (110 g/liter) and fructose (110 g/liter), 123 mg/liter of YAN, and 11 mg/liter of ammonium, was prepared according to the method of Giudici and Kunkee (60). MMM medium was used within 24 h of preparation. Fermentations were carried out in 500-ml Erlenmeyer flasks sealed with a rubber stopper and air lock with a working volume of 400 ml, similar to that described in Henderson et al. (61). In addition, the fermentations were inoculated according to the method stated in Henderson et al. (61) and likewise a volume of the inoculum to give an initial OD_600_ of ∼0.1 (∼15 ml) was added to 400 ml of MMM medium. The pH of the MMM medium was 3.25 and the fermentation temperature was maintained at 20°C. Cultures were stagnantly cultivated at 20°C for 17 days. Initial cell concentration (OD_600_) and °Bx measurements were performed at the beginning of fermentation and subsequently 10 more times over the course of the fermentation until °Bx fell below one or remained constant between two consecutive measurements. °Bx measurements were performed with a refractometer and OD_600_ measurements were performed with a spectrophotometer. Samples (10 ml) were taken at regular intervals and transferred to a 15-ml Greiner tube, sealed, and frozen at −20°C until analysis. Experiments were performed as biological triplicates.

### Cell dry weight determination.

The total cell dry weight (biomass) of the samples was determined by taking a sample directly from the culture medium and passing through dried and preweighed membrane filters with a pore size of 0.2 μm (Pall Corporation, Ann Arbor, MI, USA) by a vacuum filtration unit, as described by van Mastrigt and coworkers (62). Total biomass concentration at every sample time point was determined by transforming results from the OD_600_ measurements using a calibration curve for each yeast strain. Total cell dry weights were determined in triplicates.

### High-performance liquid chromatography analysis.

After taking a sample from the Erlenmeyer flask, cells were immediately removed by centrifugation (13,000 × *g* for 10 min at 4°C), and the supernatant was stored at −20°C until analysis. Supernatants were deproteinated by adding 0.25 ml Carrez A (0.1 M potassium ferrocyanide trihydrate) and 0.25 ml Carrez B (0.2 M zinc sulfate heptahydrate) to a 0.5-ml sample followed by centrifugation for 10 min at 13,000 × *g*. Glucose, fructose, lactate, acetate, malate, citrate, ethanol, 1-propanol, and 1,2-propanediol were quantified by high-performance liquid chromatography (HPLC) on an Ultimate 3000 (Dionex, Idstein, Germany) equipped with an Aminex HPX-87H column (300 × 7.8 mm) with precolumn (Bio-Rad), as described by van Mastrigt and coworkers (62). As the mobile phase, 5 mM sulfuric acid was used at 0.6 ml min^−1^, and the column was maintained at 40°C. The injection volume was 10 μl. Compounds were identified by a refractive index detector (RefractoMax 520) for quantification and UV measurements at 220, 250, and 280 nm for identification. All analysis was performed in duplicate.

### Ultraperformance liquid chromatography amino acid analysis.

An aliquot of 40 μl of 5-fold-diluted samples was mixed with 50 μl of 0.1 M HCl solution containing 250 μM norvaline as the internal standard. Then, 10 μl of chilled 30% sulfosalicylic acid (SSA) was added, and the solution was mixed and centrifuged (13,000 × *g*) for 10 min. at 4°C. Amino acids were derivatized using the AccQ-Tag Ultra Derivatization kit (Waters), for which 20 μl of the deproteinated sample solution supernatant or standard amino acids mixture was mixed with 60 μl of a modified AccQ-Tag Ultra Borate buffer (for deproteinated samples, 150 μl of 4 M NaOH was added to 5 ml borate buffer). Next, 20 μl of an AccQ-Tag reagent previously dissolved in 2.0 ml AccQ-Tag Ultra reagent diluent was added and vortexed for 10 s. Then, the sample solution was capped and warmed at 55°C in a heatblock for 10 min. Amino acids and ammonium were quantified by ultraperformance liquid chromatography (UPLC) on an Ultimate 3000 (Dionex, Idstein, Germany) equipped with an AccQ-Tag Ultra BEH C_18_ column (150 mm × 2.1 mm, 1.7 μm) (Waters, Milford, MA, USA) with a BEH C_18_ guard column (5 mm × 2.1 mm, 1.7 μm) (Waters, Milford, MA, USA). The column temperature was set at 55°C, and the mobile phase flow rate was maintained at 0.7 ml/min. Eluent A was 5% AccQ-Tag Ultra concentrate solvent A and Eluent B was 100% AccQ-Tag Ultra solvent B. The separation gradient was 0 to 0.04 min 99.9% A; 5.24 min 90.9% A; 7.24 min 78.8% A; 8.54 min 57.8% A; 8.55 to 10.14 min 10% A; and 10.23 to 17 min 99.9% A. One microliter of sample was injected for analysis. Compounds were detected by UV measurement at 260 nm.

The ammonium concentration was further determined and confirmed with an ammonia assay kit (Megazyme, Bray, Ireland) according to the manufacturer’s procedures. The ammonia levels were verified to exhaust after 28 h of fermentation. This method was also used to correct quantification of ammonium detected from the UPLC. Norvaline was used as an internal standard (IS) for the UPLC amino acid analysis. All analysis was performed with a single replicate.

### Volatile organic compounds analysis.

To determine the volatile organic compounds (VOCs), 2 ml of sample was transferred to a 5-ml gas chromatography (GC) vial. Samples were stored frozen (−20°C) until analysis by headspace solid-phase microextraction gas chromatography mass spectrometry (HS SPME GC-MS) (63). Samples were thawed and incubated for 5 min at 60°C with agitation. Subsequently, VOCs were extracted from the samples for 20 min at 60°C using a solid-phase microextraction fiber (50 μm Bonded Gray Hub [DVB/CAR/PDMS] Supelco, USA). The compounds were desorbed from the fiber for 10 min on a Stabilwax-DA Crossband-Carbowax-polyethylene glycol column (30 m length, 0.25 mmID, 0.5 mm df). The settings on the gas chromatograph were PTV Split-less mode 5 min at 250°C. Helium was used as carrier gas at a constant flow of 1.5 ml/min. The temperature of the GC oven was initially at 40°C. After 2 min, the temperature was raised to 240°C at a rate of 10°C/min and kept at 240°C for 5 min. Mass spectral data were collected over a range of *m/z* 33 to 250 in full scan mode with 3.0030 scans/second. VOC profiles were analyzed with Chromeleon 7.3 software. The ICIS algorithm was used for peak integration and the NIST main library was used for identification by matching mass spectral profiles with the profiles in NIST. One quantifying peak (in general the highest *m/z* peak per compound) was used per compound for quantification, while one or two confirming peaks were used for confirmation.

The 11 aroma-associated compounds studied were propan-1-ol, 3-methylbutan-1-ol (isoamylol), 2-methylpropan-1-ol (isobutanol), 2-phenylethan-1-ol, methionol, ethyl ethanoate (ethyl acetate), ethyl butanoate, ethyl hexanoate, 3-methylbutyl acetate (isoamyl acetate), 2-methylpropyl ethanoate (isobutyl acetate), and 2-phenylethyl acetate (Table 4). All of the purchased analytes had a purity of at least 98% and were purchased from Sigma-Aldrich (Sigma-Aldrich, Germany). Higher alcohols and esters were quantified using standard solutions in unfermented synthetic grape juice as similarly described in the literature (29, 64). The model solutions were spiked with various amounts of the 10 aroma compounds (higher alcohols and esters) to yield concentrations within the range of the concentrations typically found in unwooded and unaged wines (Table 4). The headspace was sampled by SPME GC-MS in the same manner as used for the model wine samples. Standard curves of each studied aroma compound were created by plotting each aroma compound's peak area against the standard concentration from the standard solutions. All samples were analyzed in duplicate.

**TABLE 4:** Summary of major of VOCs found in wines along with their chemical attributes

Compound	Chemical structure (2D)	Compound class	Concn range in wine (μg/liter)^*a*^^,^^*b*^	Organoleptic (aroma) association^*a*^^,^^*b*^
Propan-1-ol	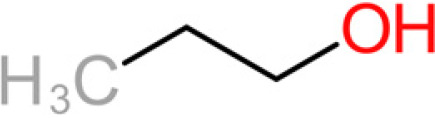	Fusel alcohol	9–68,000	Solvent, chemical
3-Methylbutan-1-ol (isoamylol)	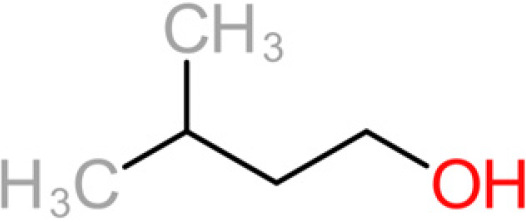	Fusel alcohol	90,000–292,000	Solvent, sweet
2-Methylpropan-1-ol (isobutanol)	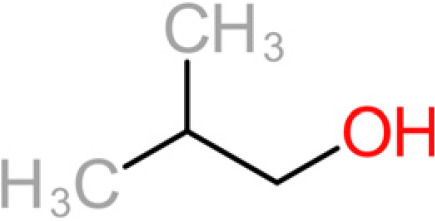	Fusel alcohol	9,000–175,000	Solvent, chemical, sweet
2-Phenylethan-1-ol (2- phenylethanol)	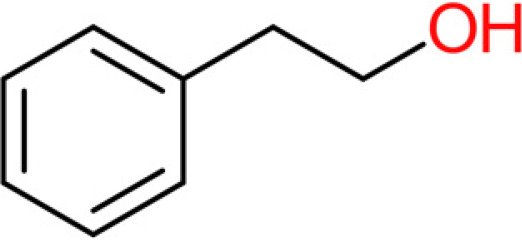	Fusel alcohol	4,000–200,000	Roses, honey, sweet
3-(Methylthio)-1-propanol (methionol)	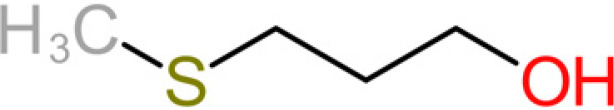	Fusel alcohol	140–5,000	Cabbage, herbal
Ethyl acetate	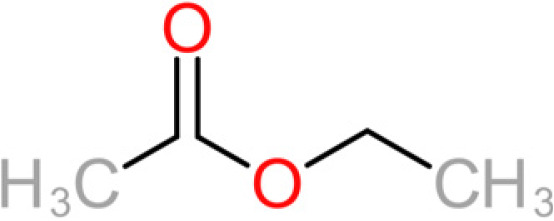	Acetate ester	2–150	Solvent, fruity, nail polish
3-Methylbutyl acetate (isoamyl acetate)	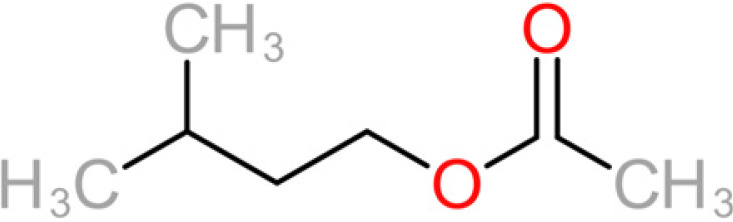	Acetate ester	115–7,400	Banana, tropical fruit
2-Methylpropyl acetate (isobutyl acetate)	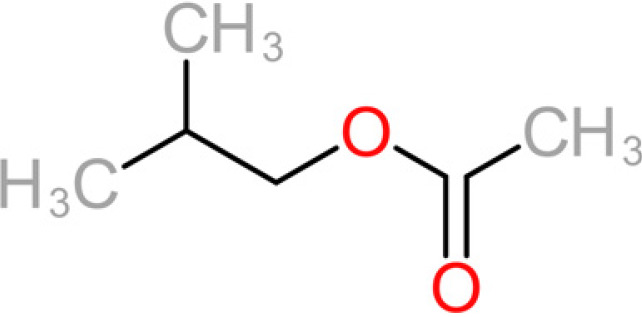	Acetate ester	40–1,600	Banana, tropical fruit
2-Phenylethyl acetate	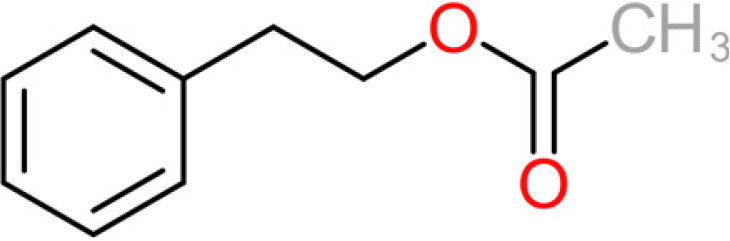	Acetate ester	0.5–750	Pear, flowery, honey
Ethyl butanoate	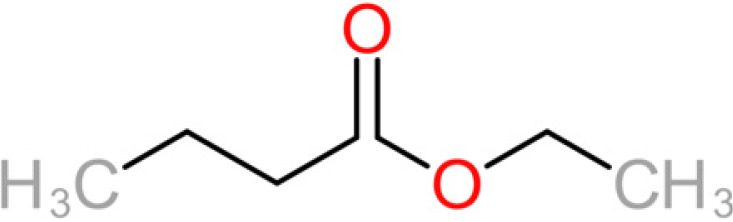	Fatty acid ethyl ester	70–2,200	Floral, fruity
Ethyl hexanoate	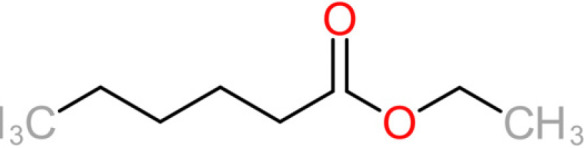	Fatty acid ethyl ester	150–2,800	Green apple, unripe fruit

a Ribéreau-Gayon et al. (39).

b Swiegers and Pretorius (28).

### Multivariate data and statistical analyses.

The yeast strain-specific impact on the kinetics of amino acid consumption and aroma compound formation for the 11 volatile aromas was determined by plotting the compound concentrations throughout the fermentation. Furthermore, several performance metrics were accessed from the dynamic consumption and production profiles, such as the final concentration of volatile aroma compounds and utilization of nitrogenous compounds. In this study, nitrogen (ammonia/amino acids) utilization was determined as the difference in the ammonia/amino acid concentration from beginning to sample time point of interest throughout the fermentation (29). The final volatile aroma concentration was determined at 404 h, when all of the fermentations were finished. Principal-component analysis (PCA) was performed using R (version 3.6.2, R Core Team, 2020).

One-way analysis of variance (ANOVA) (significance level of *P* < 0.05) was performed in R (version 3.6.2) using the statistics package applying post hoc Tukey’s honest significant difference (HSD) test (significance level of *P* < 0.05), as well as in SPSS (IBM SPSS Statistics 27, IBM Corp., Armonk, NY).

A multivariate statistical method known as partial least-squares (PLS) regression was employed to determine a relationship between biomass and VOC production data (Y-Block) and nitrogen (ammonia and amino acid) utilization data (X-Block). The utilization was calculated as the difference in concentration at the beginning of the fermentation to concentration at the sampled time point. These two data blocks were imported into MATLAB (version MATLAB [2017b], MathWorks, Natick, MA) for PLS regression analysis and interval-PLS (iPLS) variable selection using the PLS Toolbox (version 8.9; Eigenvector Research, Inc., Manson, WA). Parameters for iPLS were selected according to the method of Wise et al. (65) and Anderson and Bro (66) and adapted from the technique of Henderson et al. (61). Reverse-analysis-mode iPLS selection was conducted with an interval size of one variable with a maximum of 18 latent variables (LVs). The step size, which is the space between interval centers, and number of variables chosen were automatically decided based on when there was no improvement in the root mean squared error in cross-validation (RMSECV).

Subsequently, once iPLS was completed, the selected variables were added to PLS Toolbox for structured equation modeling. The approach used was adapted based on an approach by Henderson et al. (61) using the SIMPLS algorithm (67) at a confidence limit of 0.95. Auto-scaling followed by mean centering was applied to the preprocessing of the X- and Y-blocks. The PLS model was cross-validated using Venetian Blinds with 24 data splits and a maximum of 18 LVs. The quality and predictive capability of the PLS model were evaluated from the correlation coefficient (*R*^2^), cross-validated correlation coefficient (*Q*^2^), root mean squared error of correlation (RSMEC), and RMSECV. The impact of selected nutrient utilization variables on the PLS model was assessed using scores, loadings, and regression vector plots. Overall, the PLS model was used to determine the nutrient utilization (ammonia and amino acids) which contribute to the biomass formation and VOCs production during fermentation.

### Metabolic modeling.

For model analysis, we used the latest genome-scale metabolic model (GSMM) of S. cerevisiae, Yeast 8.4.2 (https://github.com/SysBioChalmers/yeast-GEM) (68). To adequately model an anaerobic fermentation, we proceeded as suggested by Heavner et al. (69), constraining $v_{O_{2}}$ to zero (LB = UB = 0 [mmol/g dry weight per h]), allowing unrestricted uptake of ergosterol (r_1757), lanosterol (r_1915), zymosterol (r_2106), 14-demethyllanosterol (r_2134), and ergosta-5,7,22,24(28)-tetraen-3beta-ol (r_2137) and oleate (r_2189). In addition, pathways, including the oxaloacetate-malate shuttle and glycerol dehydrogenase reaction, were unrestricted as described by Sanchez et al. (70, 71) (in the model this was achieved by blocking reactions r_0713, r_0714, and r_0487). Heme A was also removed from the biomass equation, as it is not used under anaerobic conditions. Expanded coverage of the Ehrlich pathway and accompanying reactions and metabolites related to sulfur reduction and ester synthesis were added to Yeast 8 (72). Experimentally derived net uptake and production fluxes, which were taken from measured data from the four yeast strains during exponential growth phase time points 10 h, 24 h, and 36 h, (see Fig. S3 in the supplemental material), were used to constrain the model in the form of exchange reactions (LB = UB) for the sugars, amino acids, organic acids, VOCs, and other by-products.

We applied parsimonious flux balance analysis (pFBA) to evaluate which reactions and pathways are essential under the given system conditions and eliminate blocked reactions (i.e., reactions that cannot carry a flux under any condition). It uses a bilevel optimization in which the growth rate (biomass) is optimized using FBA, followed by the minimization of total flux through all gene-associated reactions at the maximum growth rate calculated. The underlying assumption is that, under growth pressure, there is a selection for strains that can reach the highest growth yield while using the minimum amount of enzyme. Then, genes/reactions are classified into six categories based on Lewis et al. (73), i.e., (i) essential genes, metabolic genes necessary for growth in the given media; (ii) pFBA optima, nonessential genes contributing to the optimal growth rate and minimum gene-associated flux; (iii) enzymatically less efficient (ELE), genes requiring more flux through enzymatic steps than alternative pathways that meet the same predicted growth rate; (iv) metabolically less efficient (MLE), genes requiring a growth rate reduction if used; (v) pFBA no-flux, genes that are unable to carry flux in the experimental conditions; and (vi) blocked, genes that are only associated with the reactions that cannot carry a flux under any condition (“blocked” reactions). Flux enrichment analysis (FEA) was applied to test whether subsystems/metabolic pathways have significantly more essential genes, hence enriched, than expected by chance. The statistical significance for the pathways or reaction subsystems to be enriched contained *P* < 0.01. All reactions, metabolites, and subsystems are defined according to the Biochemical Genetic and Genomic (BiGG) database convention (74). Results on pFBA and FEA as reported in the results section are derived from the experimental data of strain Uvaferm at time point 24 h and it must be noted that the results are independent of the strain (see the supplemental material text file for the entire essential reaction list).

All metabolic modeling was performed in MATLAB (version MATLAB [2017b], MathWorks, Natick, MA) using the Cobra Toolbox 3.0 (75). For instance, the specific functions used for pFBA and FEA are documented in the following tutorial links: https://opencobra.github.io/cobratoolbox/stable/tutorials/tutorialPFBA.html and https://opencobra.github.io/cobratoolbox/stable/modules/analysis/fluxEnrichmentAnalysis/index.html.

### Data availability.

All data generated or analyzed during this study are included in this published article and its supplemental material.
