# Supplementary material for: Nitrogenous Compound Utilization and Production of Volatile Organic Compounds among Commercial Wine Yeasts Highlight Strain-Specific Metabolic Diversity
Source: Microbiol Spectr. 2021 Jul 21;9(1):10.1128/spectrum.00485-21. doi: 10.1128/spectrum.00485-21 (PMC8562342; doi:10.1128/spectrum.00485-21)
Supplement: SUPPLEMENTAL FILE 1 — Supplemental material. Download SPECTRUM00485-21_Supp_1_seq8.docx, DOCX file, 0.08 MB [file spectrum00485-21_supp_1_seq8.docx]

**
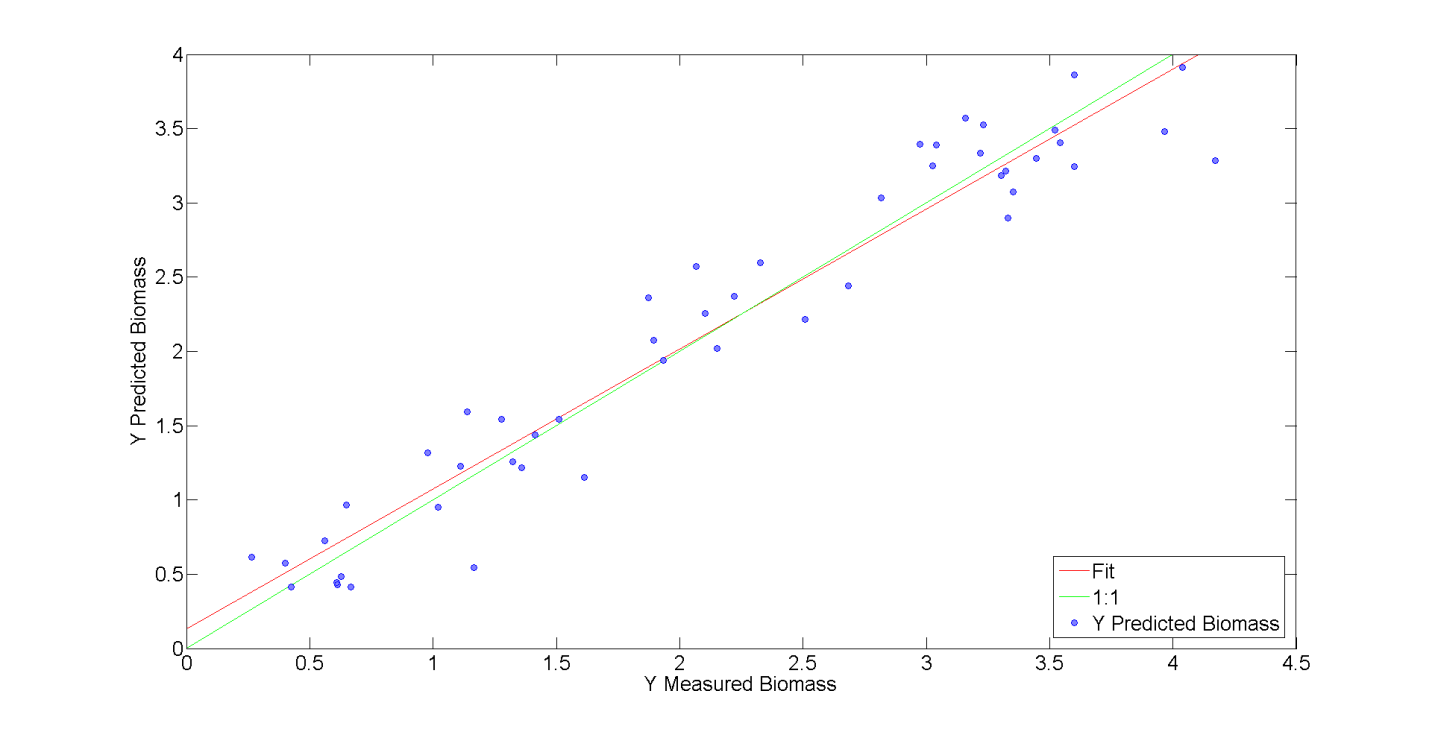
**

**
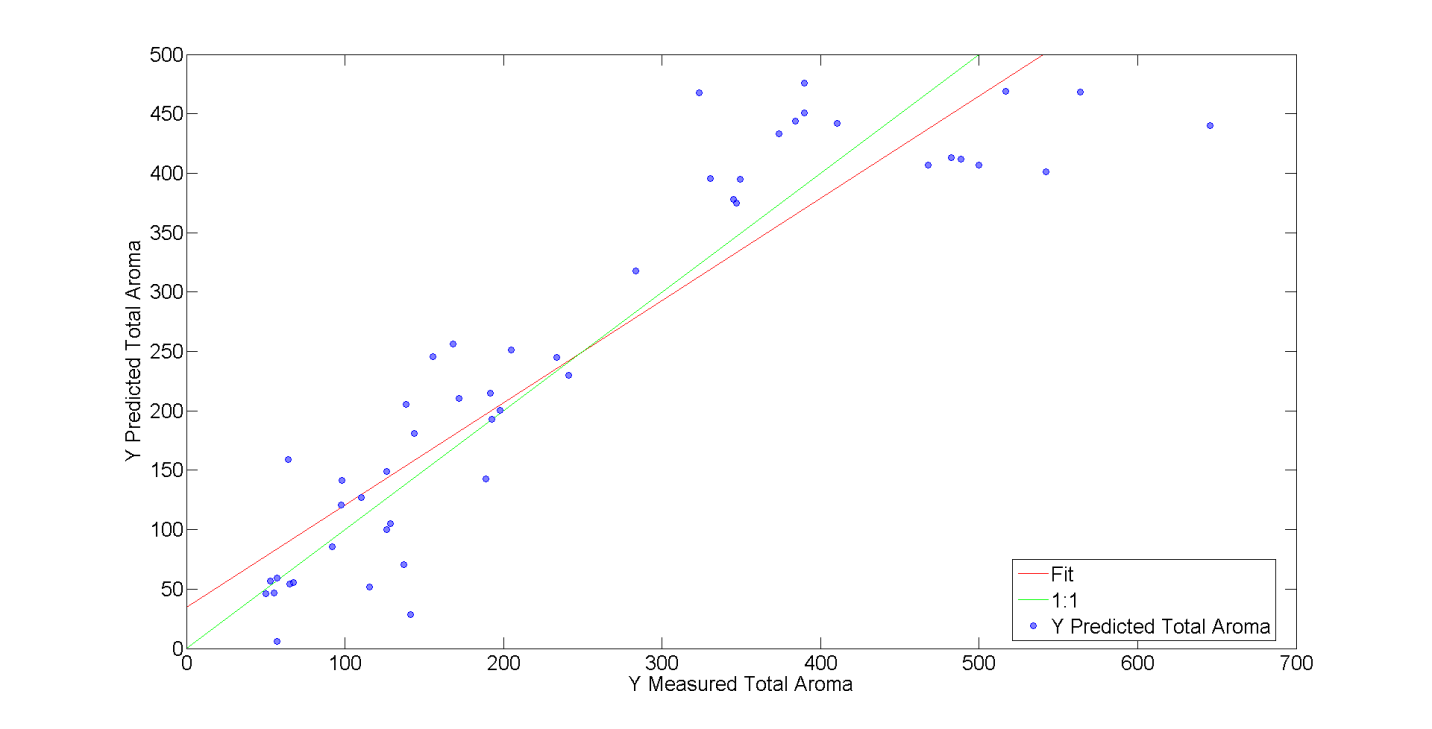
**

**FIG S1** Partial least-squares regression modeling for all strains correlating amino acid and nitrogen utilization variables to the fermentation response variables for biomass concentration (A) and total aroma concentration (B). The partial least-squares linear regression fit illustrates the measured versus predicted Y-block parameter. The linear regression equation for the model for panel A is Y = 0.91X + 0.87and for panel B is Y = 0.86 X +0.15 such that Y = Y predicted and X = Y measured.

Biomass

**
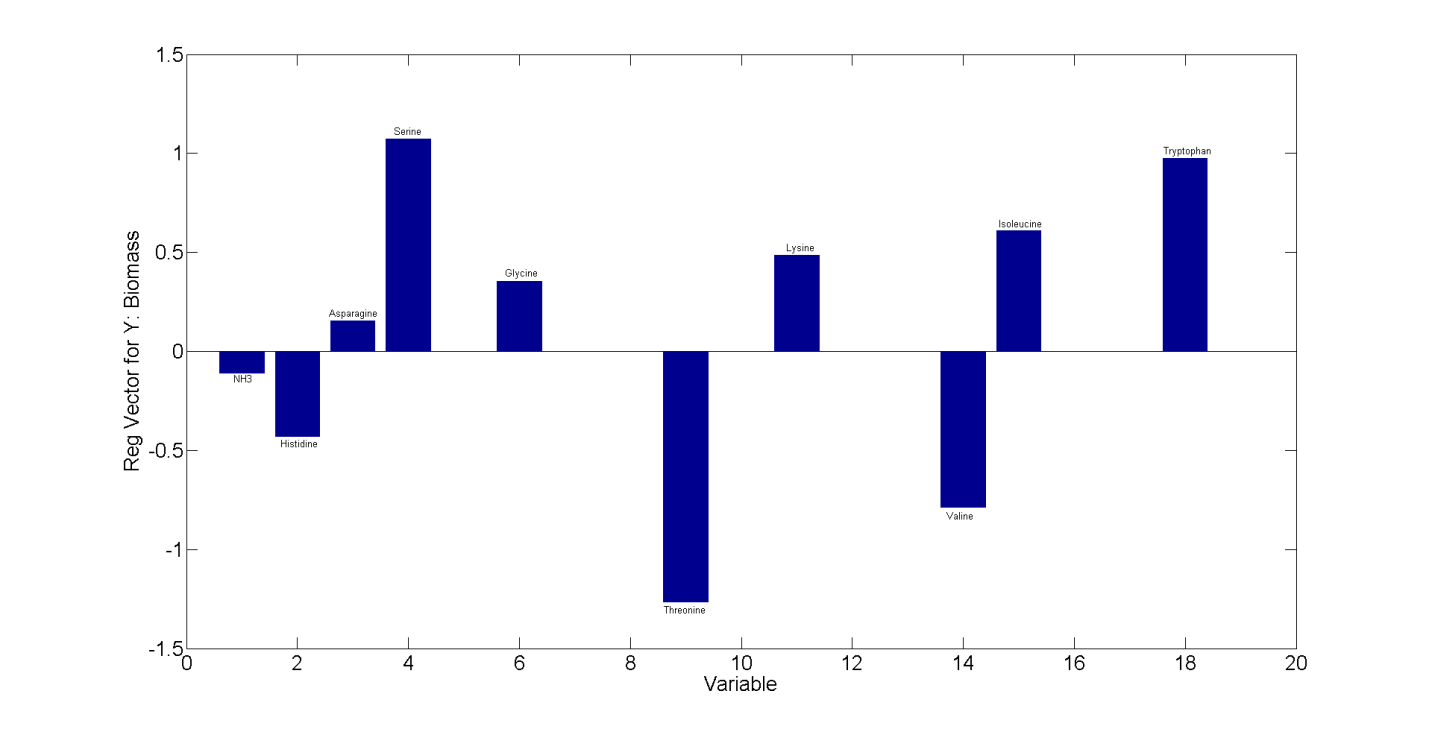
**

Total Aroma


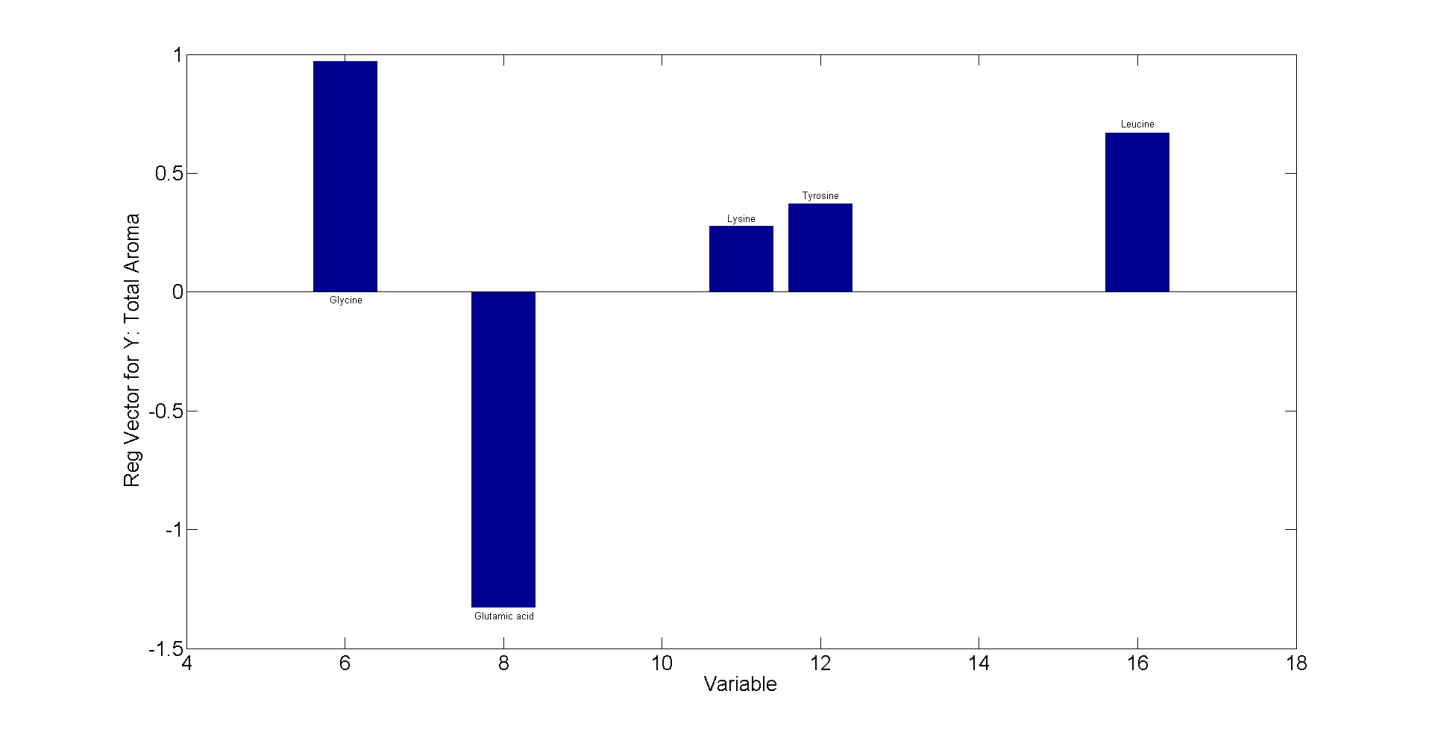


Propan-1-ol


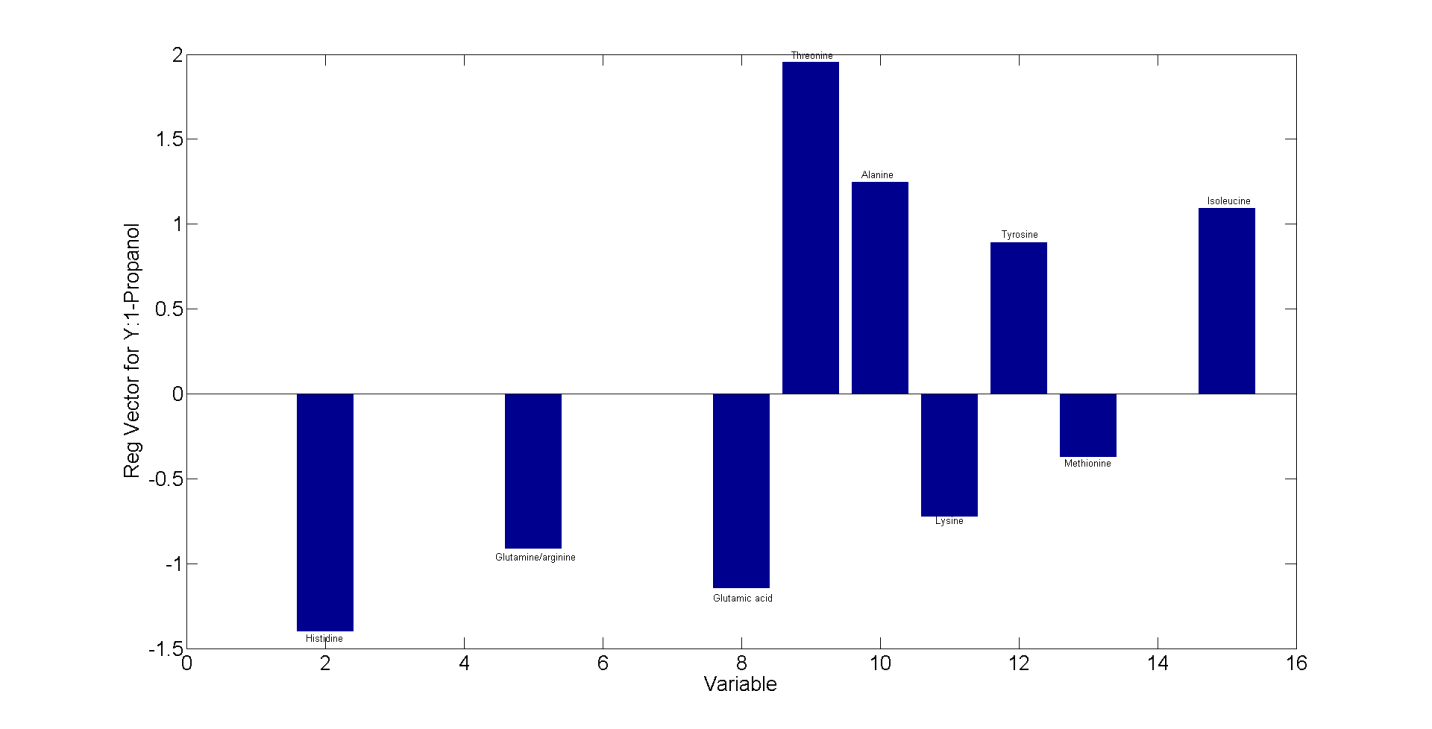


3 Methyl-1-butanol


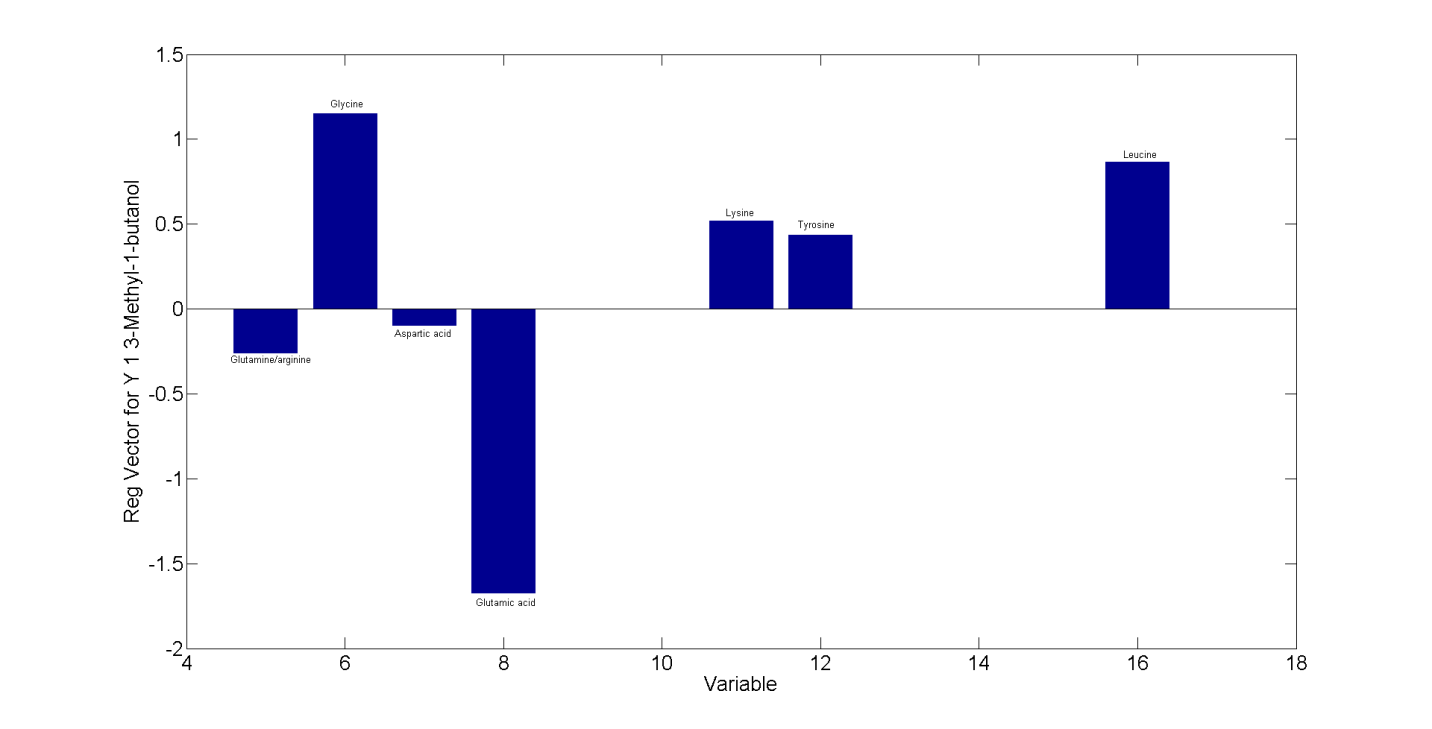


2-Methylpropan-1-ol


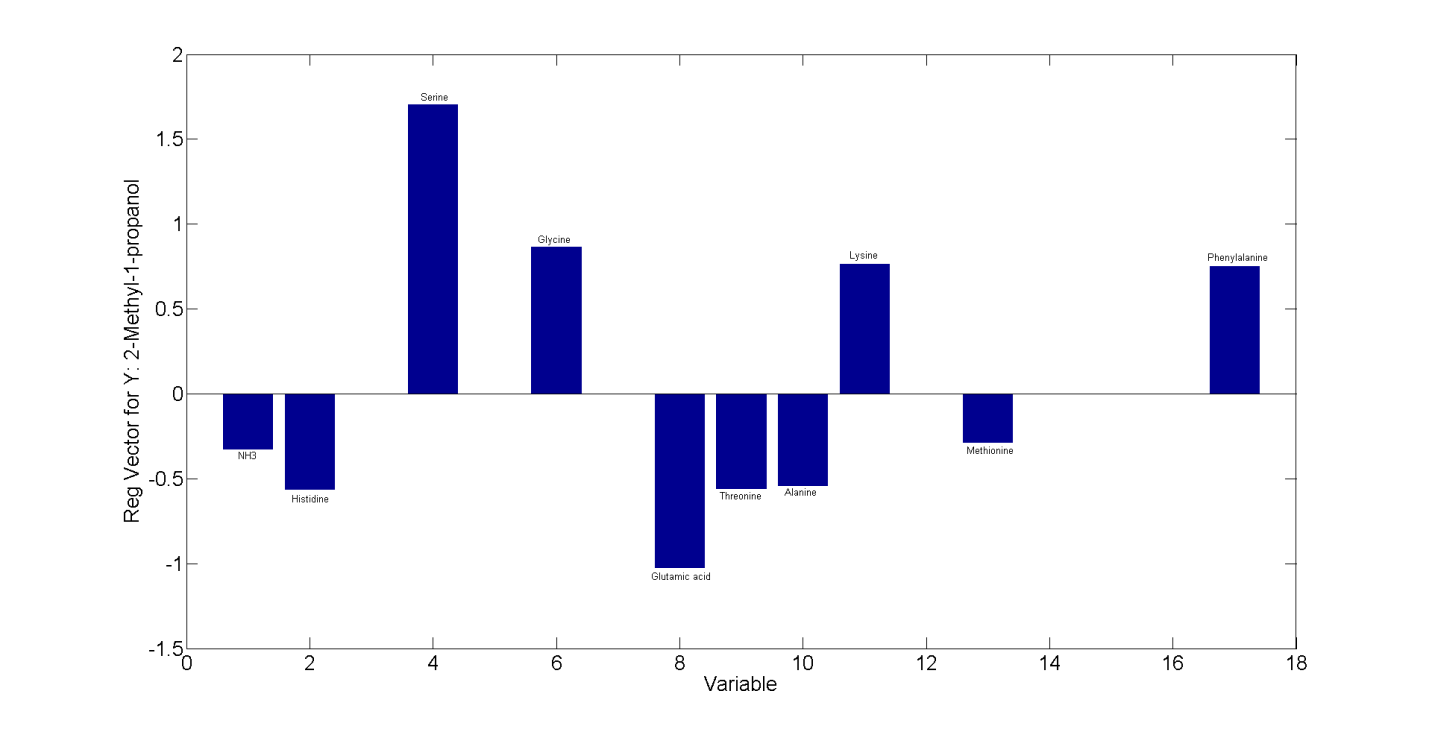


2-Phenylethan-1-ol


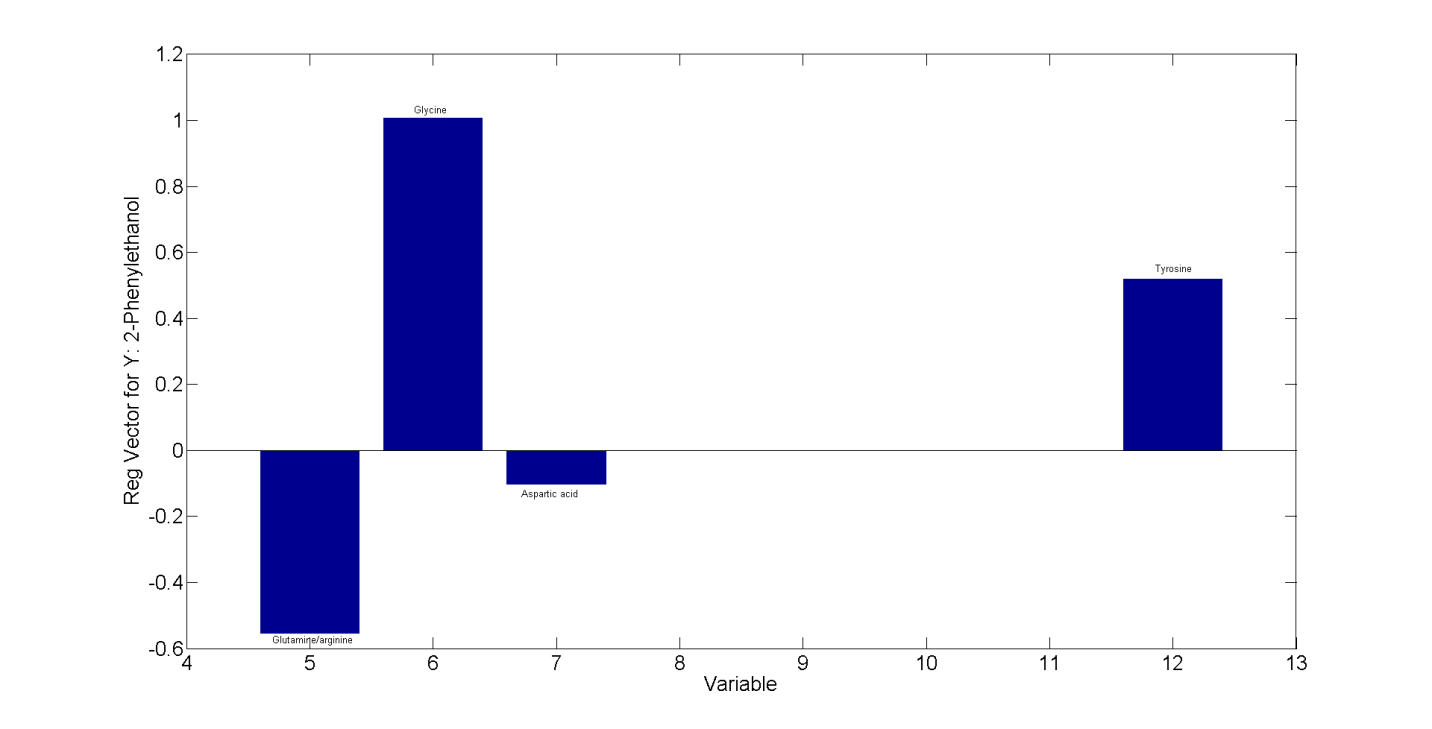


Methionol


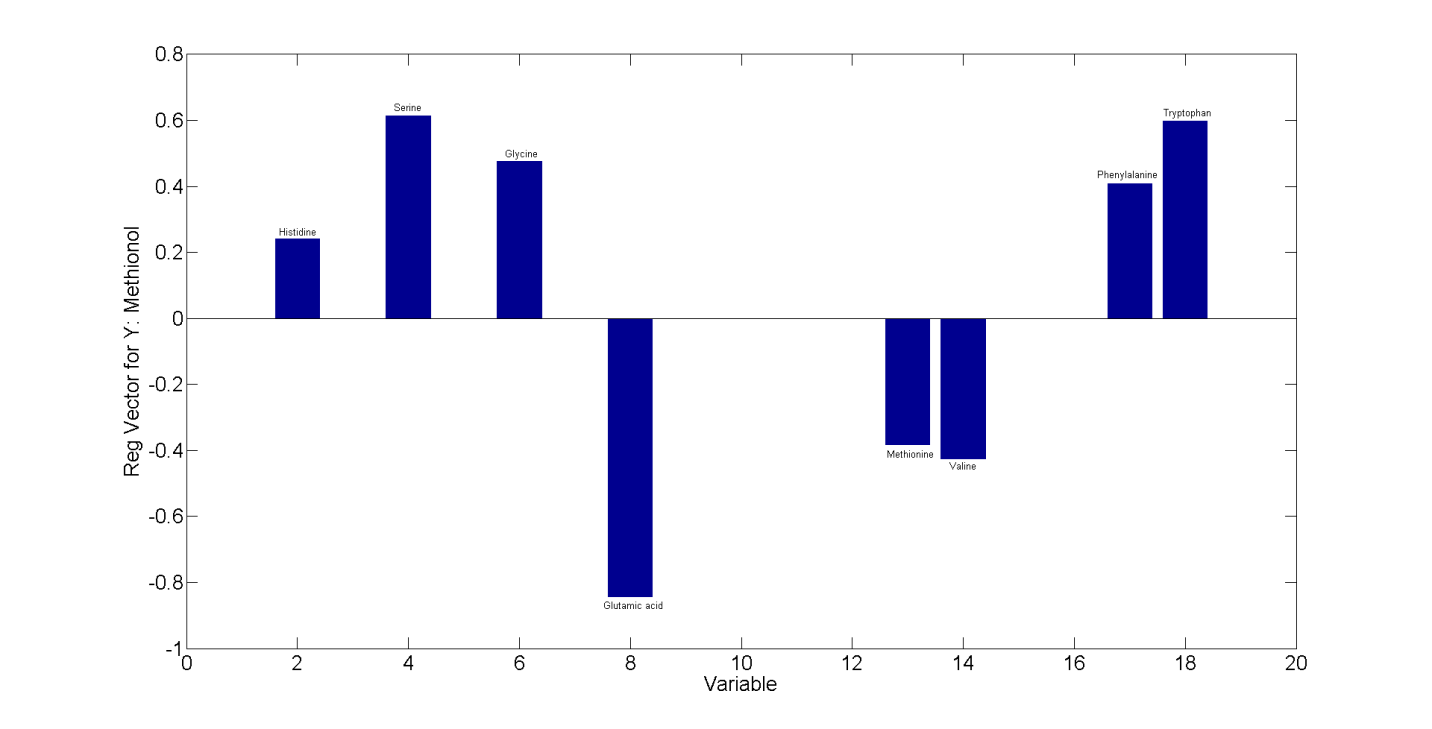


Ethyl acetate


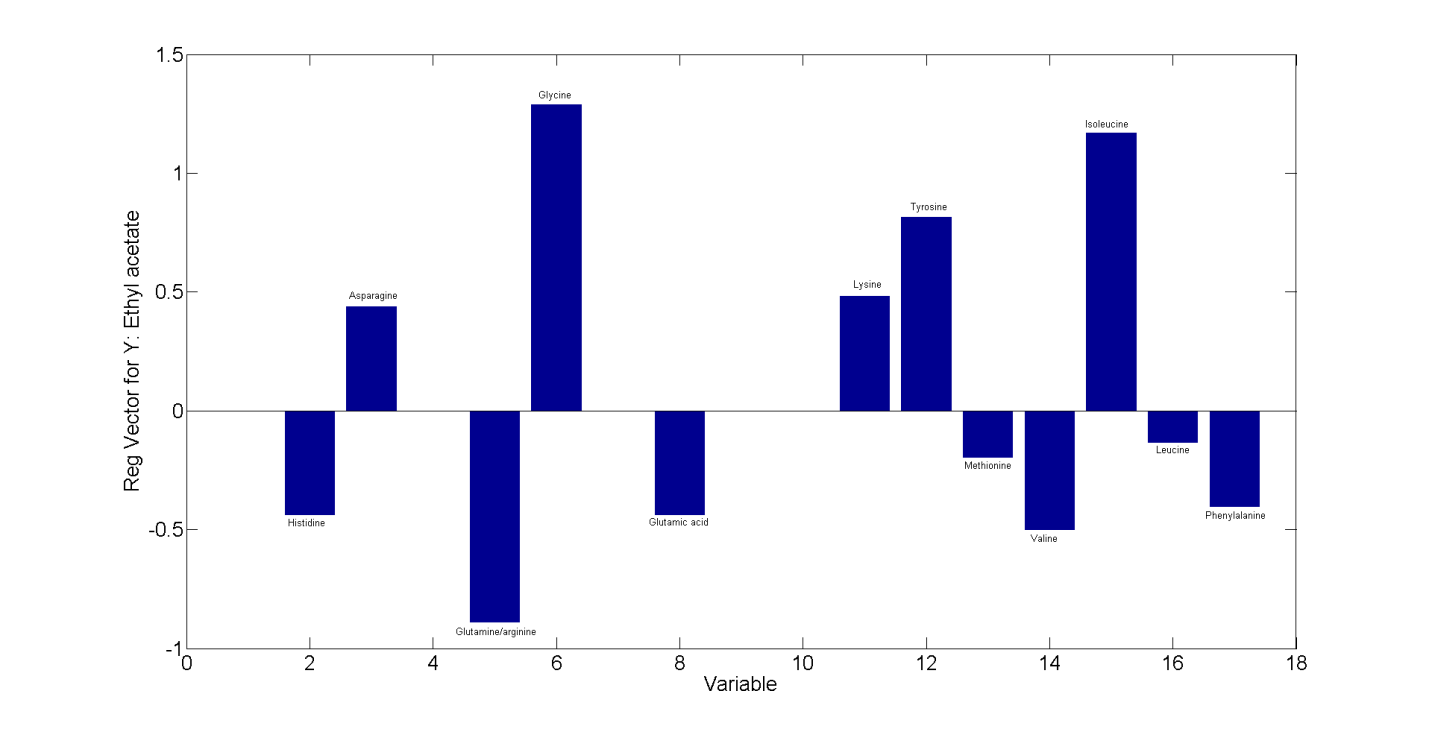


3-Methylbutyl acetate


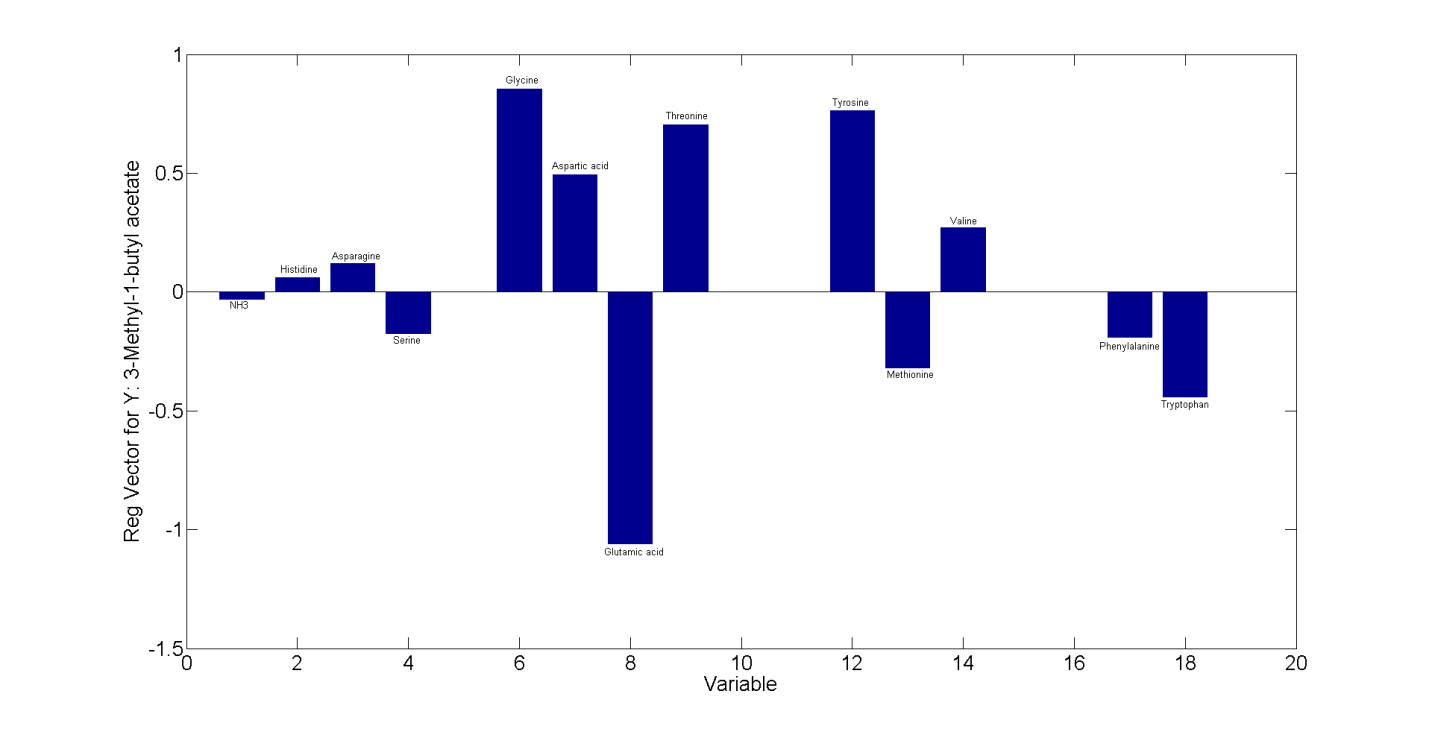


2-Methylpropyl acetate


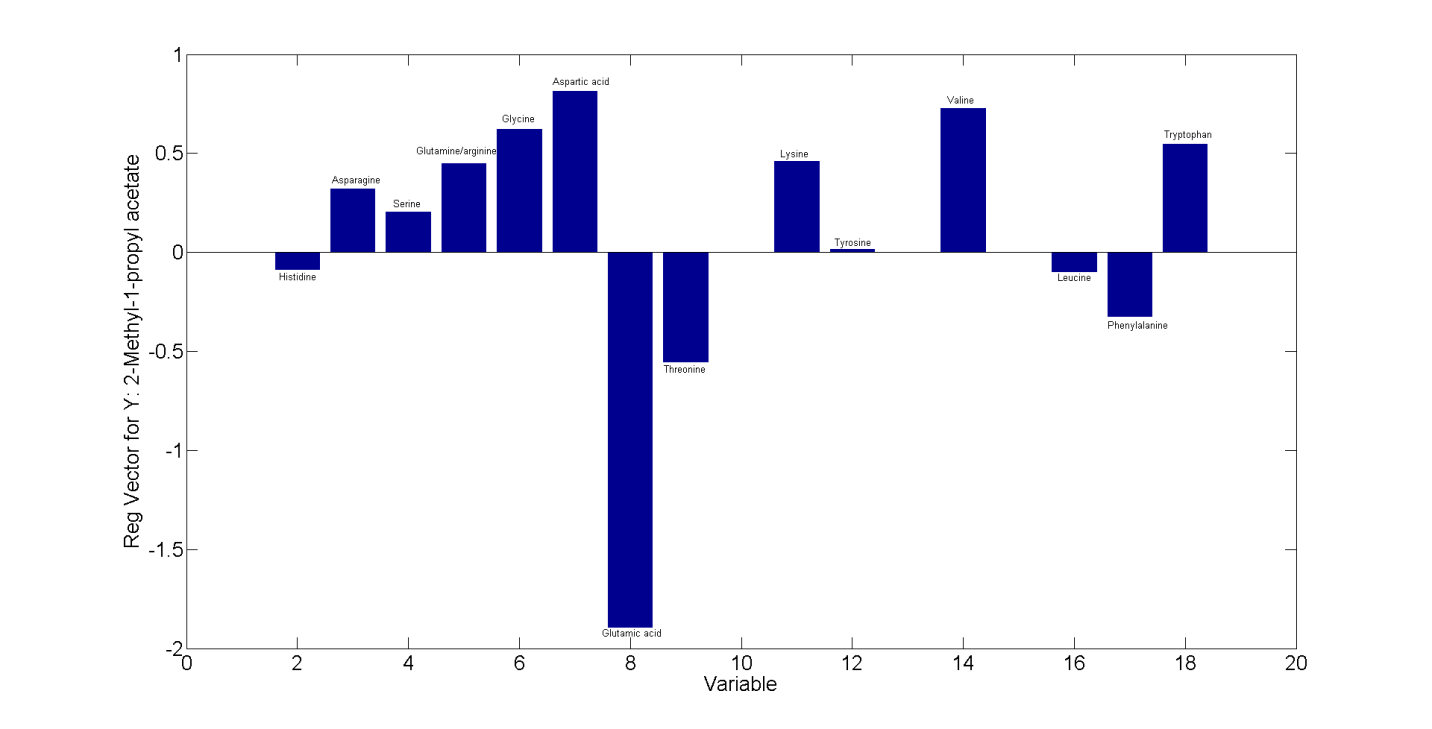


2-Phenylethyl acetate


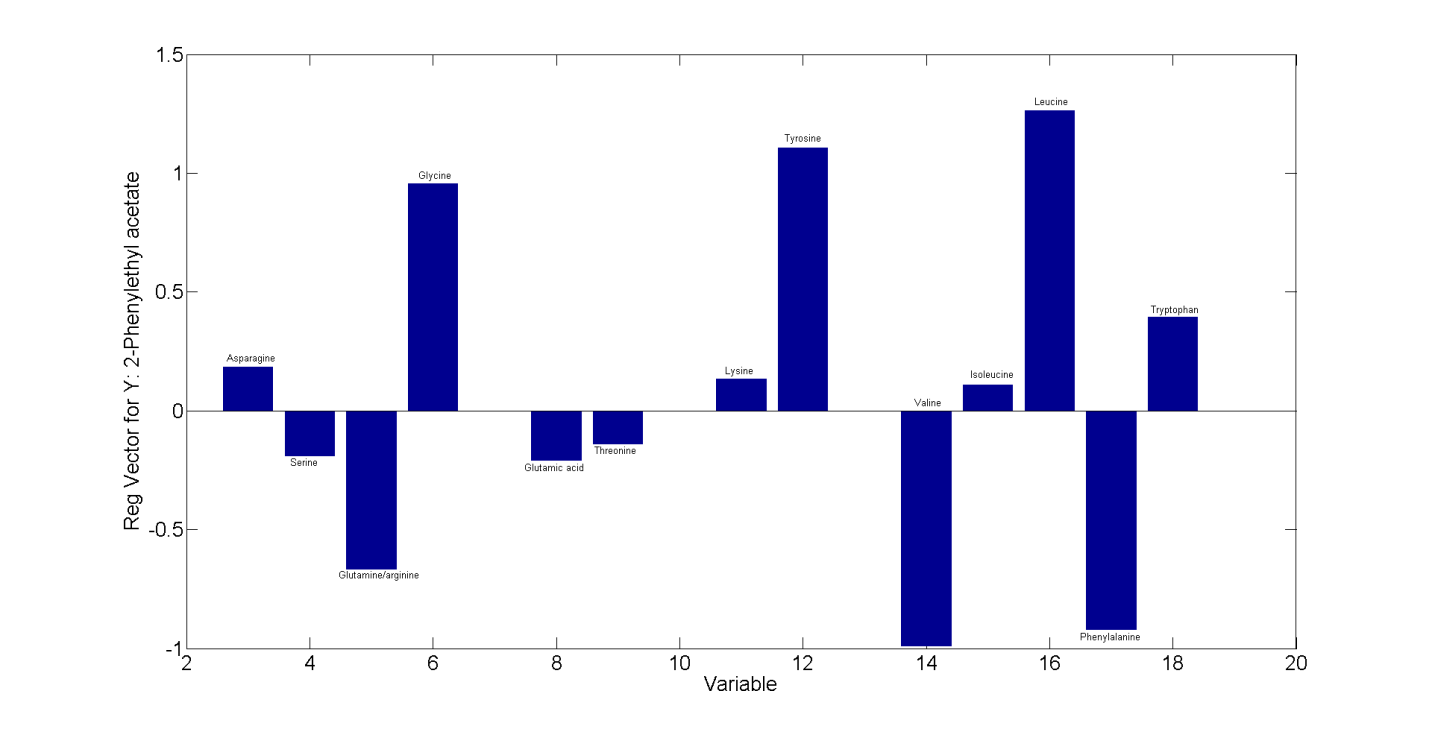


Ethyl butanoate


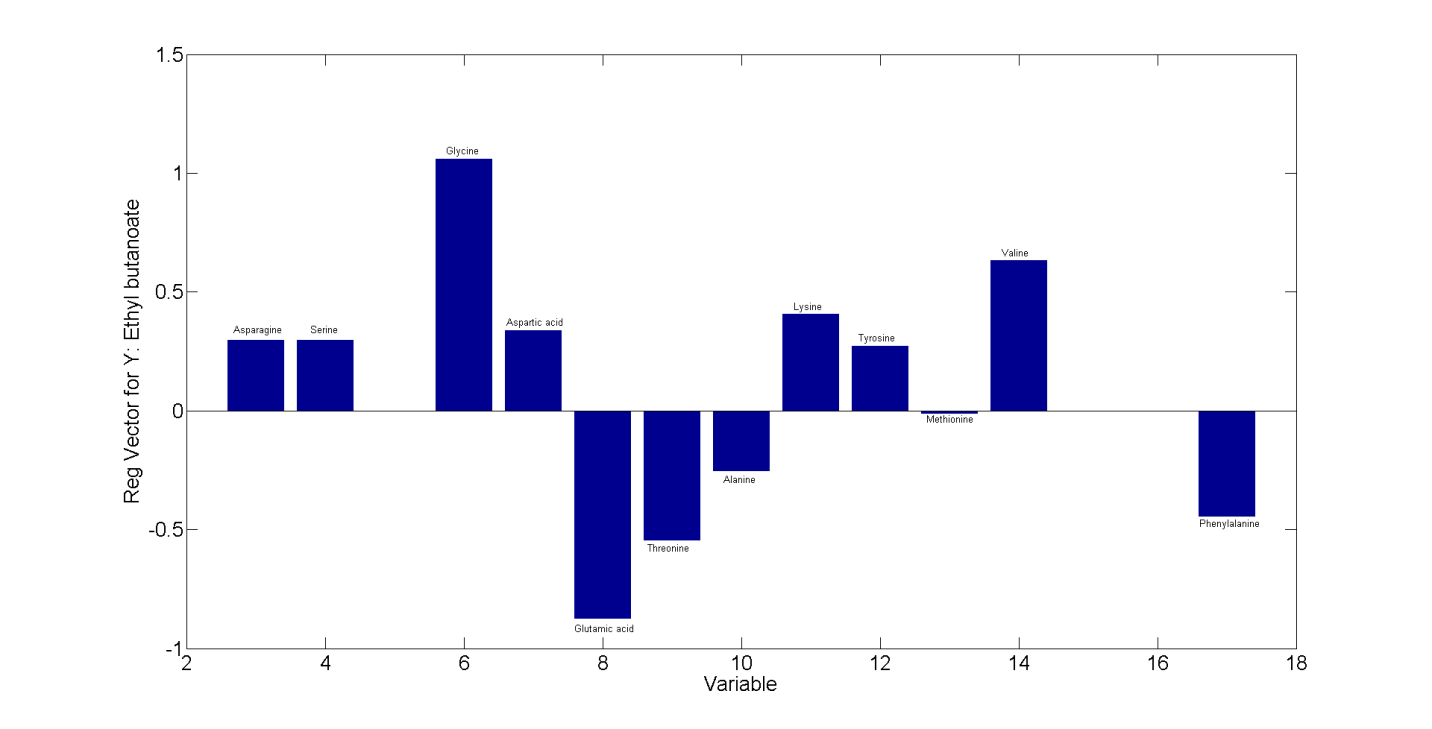


Ethyl hexanoate


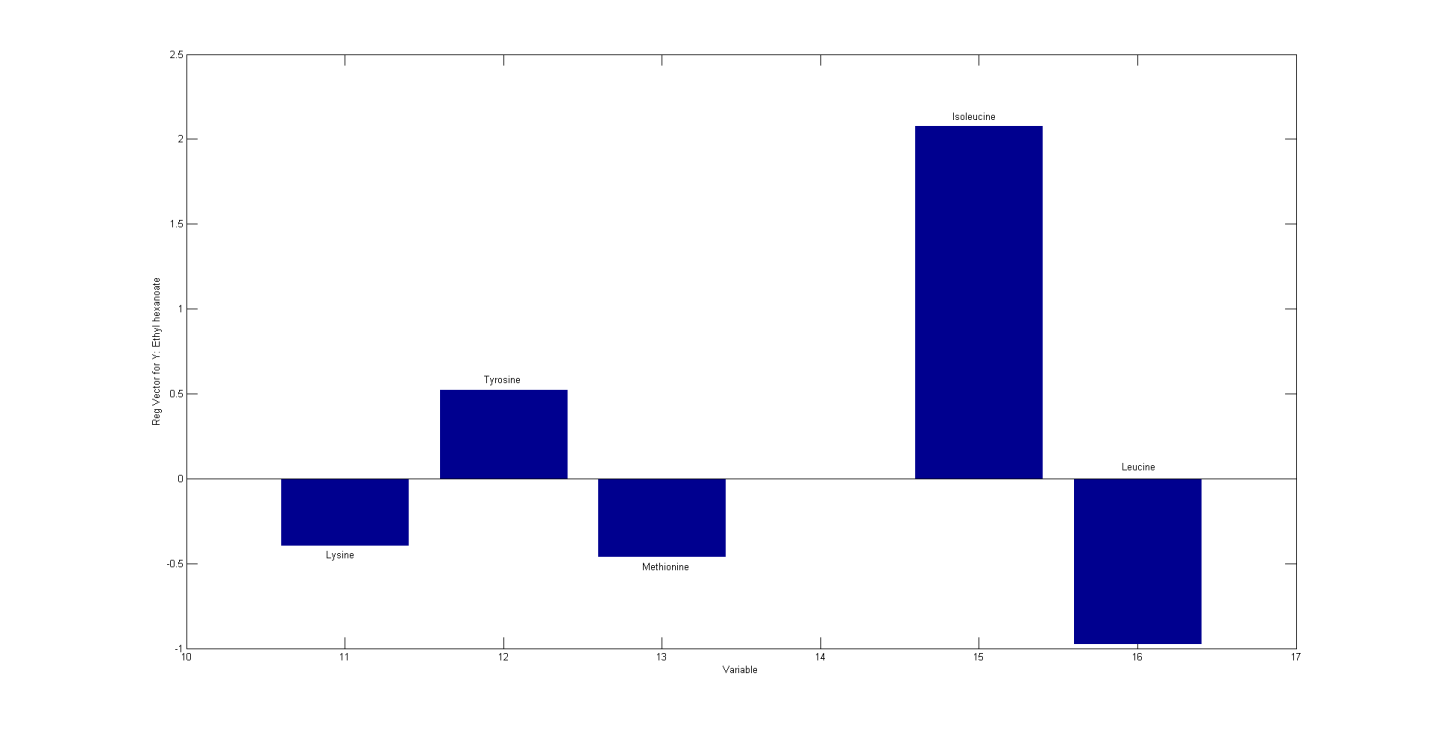


Fusel alcohols


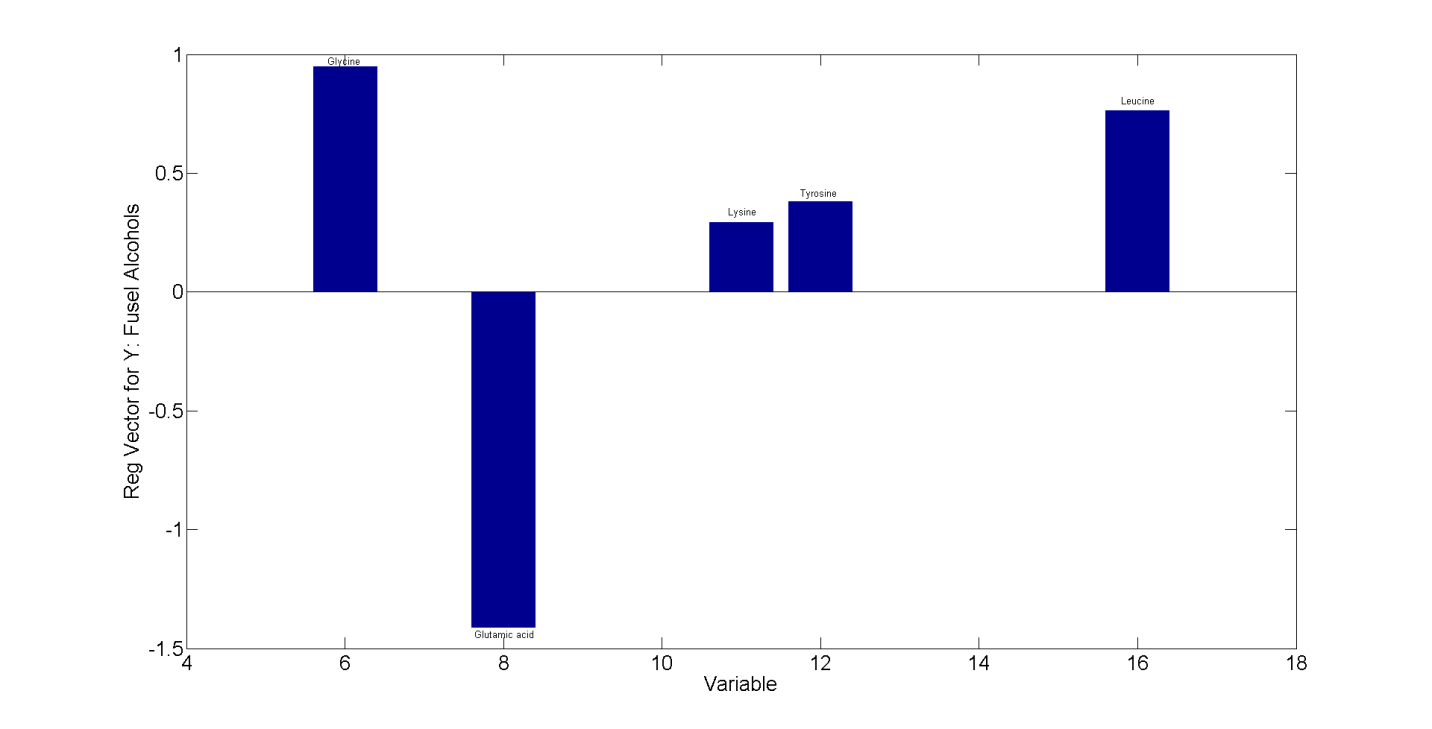


Acetate Esters


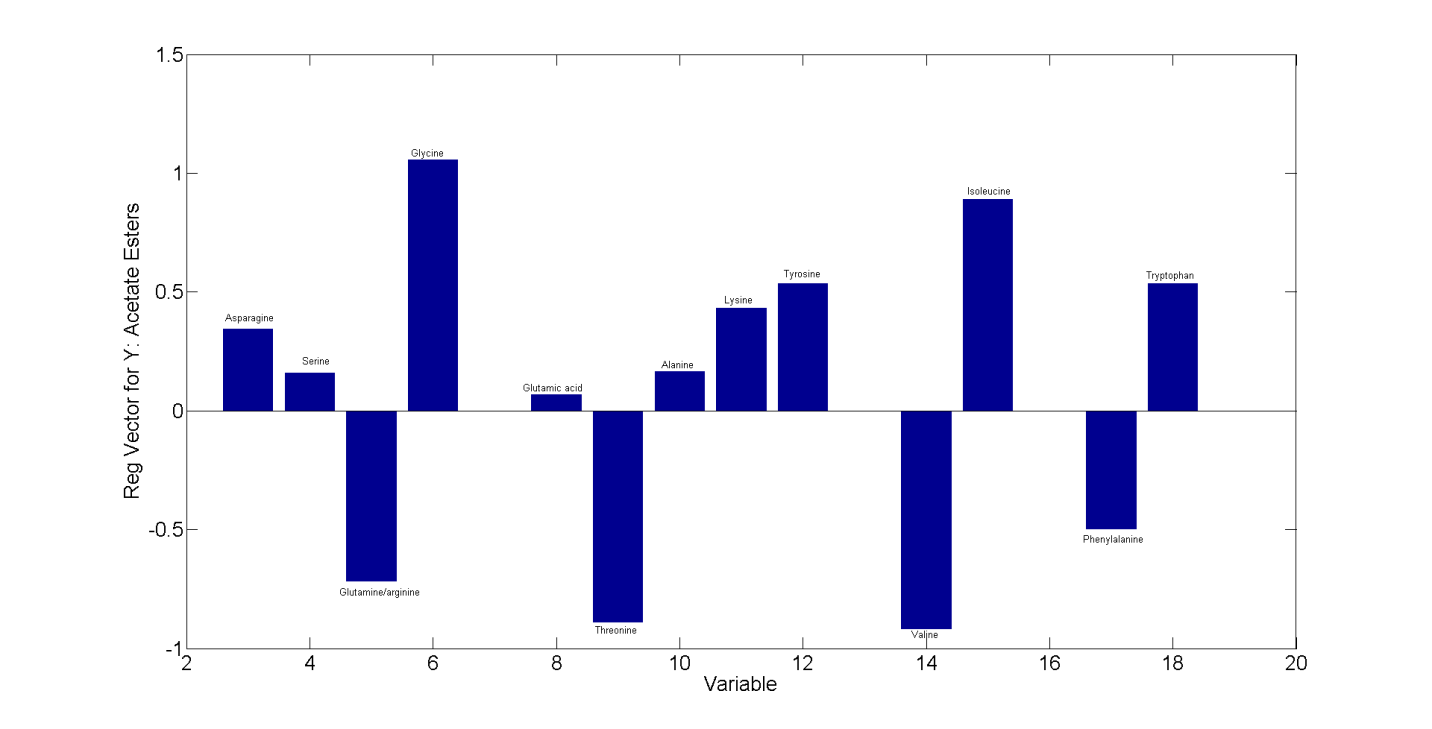


Ethyl Esters


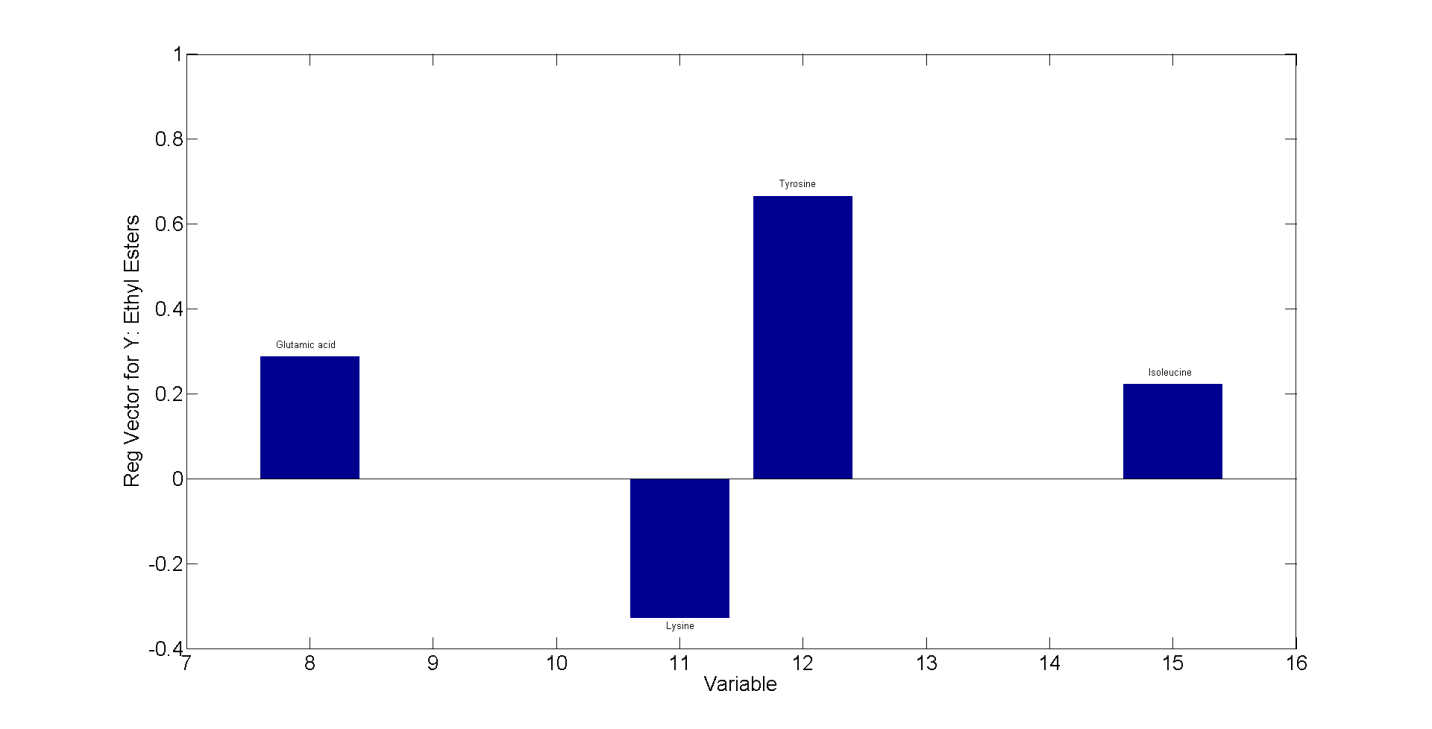


**FIG S2** Regression vector plot for respective models for all strains, indicating the relationship between yeast strains and nitrogen (amino acid/ammonia) utilization indicating each nitrogenous compound’s contribution (i.e., weight) to the PLS regression model. Positive values for the variable indicate a positive correlation between the predictor and response variables, whereas negative values indicate a negative correlation between the predictor and response variable


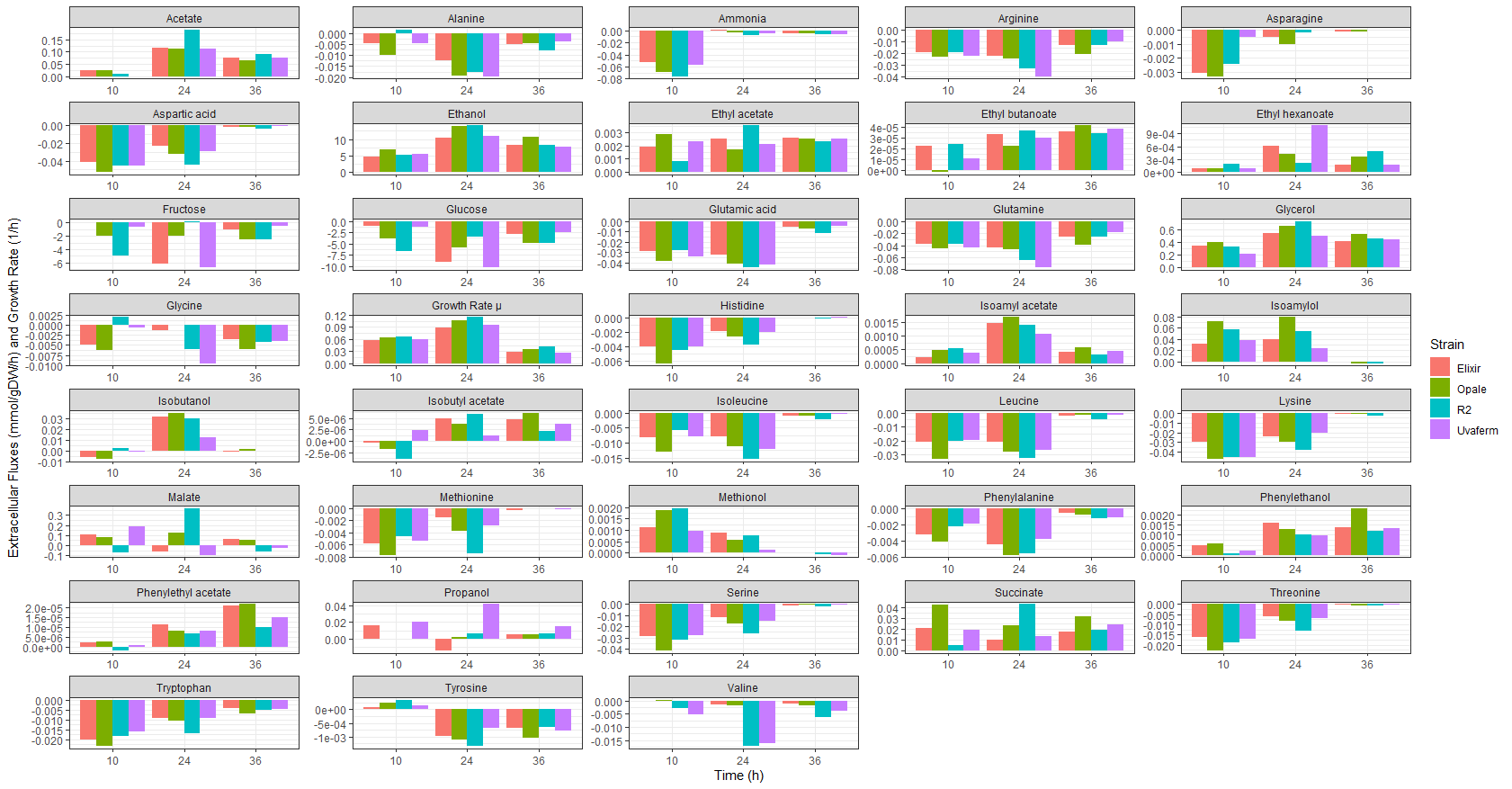
**FIG S3** Bar chart of the fluxes used as constraints for Parsimonious Flux Balance Analysis(pFBA) and Flux Enrichment Analysis (FEA). All during exponential growth phase.
